# UHRF1-mediated HIF-1α stabilization promotes ovarian cancer through metabolic reprogramming and angiogenesis

**DOI:** 10.1038/s41419-025-08033-w

**Published:** 2025-10-24

**Authors:** Yuanna Jiang, Fang Peng, Yibo Chen, Aoyu Fu, Ruichen Yang, Ziyue Yang, Qian Wang, Lanqin Cao

**Affiliations:** 1 Department of Gynecology, Xiangya Hospital, Central South University, Changsha, China; 2 National Health Commission (NHC) Key Laboratory of Cancer Proteomics, Xiangya Hospital, Central South University, Changsha, China; 3 Hunan Provincial Key Laboratory of Regional Hereditary Birth Defects Prevention and Control, Changsha Hospital for Maternal, Changsha, China

**Keywords:** Ovarian cancer, Tumour biomarkers, Ubiquitylation, Oncogenes

## Abstract

Ubiquitin-like PHD and RING finger domain-containing protein 1 (UHRF1) is an important epigenetic regulatory factor that is highly expressed in various cancers and participates in tumorigenesis and progression. However, the role and molecular mechanisms of UHRF1 in ovarian cancer (OC) remain unclear. Through survival analysis, cellular functional experiments, and animal studies, we identified UHRF1 as a key gene influencing OC progression and prognosis. Hypoxia-inducible factor-1 (HIF-1α), a well-known pro-cancer molecule, undergoes classic degradation via the ubiquitin–proteasome pathway. We discovered that UHRF1 interacts with HIF-1α, affecting its hydroxylation level, thereby inhibiting HIF-1α polyubiquitination and degradation. Functional experiments revealed that knocking down HIF-1α in stable UHRF1-overexpressing cell lines significantly reversed the malignant phenotype of OC cells. Furthermore, UHRF1 can also regulate the expression of key downstream molecules such as GLUT1, HK2, LDHA, and VEGFA by modulating HIF-1α, thus influencing tumor cell metabolism and angiogenesis. In summary, our findings suggest that UHRF1 plays a crucial role in the development of OC by regulating the expression of HIF-1α.

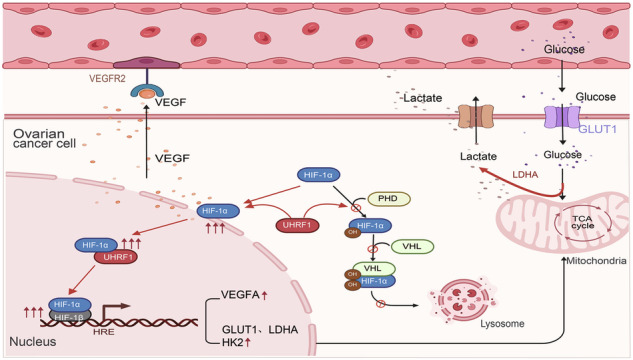

## Introduction

Ovarian cancer (OC) is one of the three most malignant tumors affecting women’s health, with high incidence and mortality rates globally [1]. Historically, OC was believed to originate from the ovarian surface epithelium [2]. However, recent studies, including those from the National Cancer Institute, suggest that OC may instead arise from the fallopian tube [3, 4]. As a result, our study included both normal ovarian tissue (NOT) and normal fallopian tube tissue (NFT) as controls. As a rapidly progressing and highly malignant solid tumor, OC has an elevated demand for oxygen and nutrients. Previous studies have shown that tumor cells undergo metabolic alterations, known as metabolic reprogramming, to cope with various environmental stressors [5]. Metabolic reprogramming-related drivers can autonomously regulate cellular metabolism, and the aberrant activation of these drivers enables cells within nascent tumors to acquire metabolic traits that support cell survival, immune evasion, and proliferative growth [6]. A growing body of evidence suggests that metabolic reprogramming is a major contributor to the failure of overall therapeutic efficacy in cancer treatment [7]. In addition, tumor angiogenesis plays a crucial role in the progression of OC, and robust angiogenic capabilities are key to the rapid growth and metastasis of OC [8].

Ubiquitin-like PHD and RING finger domain-containing protein 1 (UHRF1) is an important epigenetic regulator involved in modulating various chromatin modification proteins [9]. UHRF1 participates in different epigenetic processes through its distinct domains. The tandem Tudor domain (TTD) allows UHRF1 to bind to H3R2 and H3K9me2/3, whereas the plant homeodomain (PHD) determines its binding specificity. The SRA domain interacts with DNA methyltransferase 1 and histone deacetylase 1, facilitating DNA methylation and histone modifications [10**–**12]. In lung adenocarcinoma, UHRF1 overexpression can promote cell proliferation and dedifferentiation [13]. Studies have shown that downregulation of UHRF1 leads to cell cycle arrest at the G1/S transition and can induce caspase-8-dependent apoptosis [14]. However, the function of UHRF1 extends beyond this, and previous studies have indicated that UHRF1 is also involved in the regulation of tumor metabolism [15**–**17]. The oncogenic role of UHRF1 has been confirmed in various types of tumors. Nevertheless, the role of UHRF1 in OC remains poorly understood. This study revealed that in OC, UHRF1 promotes metabolic reprogramming and angiogenesis by inhibiting hypoxia-inducible factor 1 α (HIF-1α) degradation, thereby activating the HIF-1 pathway and driving OC progression.

HIF-1 is a major regulator of oxygen homeostasis [18] and consists of a heterodimeric DNA-binding complex composed of two basic helix–loop–helix proteins from the PAS family. These subunits are constitutive of HIF-1β and hypoxia-inducible HIF-1α [19]. HIF-1 is involved in regulating multiple pathways, including metabolic reprogramming, erythropoiesis, angiogenesis, cell growth and differentiation, and apoptosis, and is a critical factor in development, physiology, and disease [20]. The activity of HIF-1 is regulated by the stability of HIF-1α. Under normoxic conditions, the hydroxylation of proline residues or acetylation of lysine residues in the oxygen-dependent degradation domain (ODDD) of HIF-1α triggers its binding to the von Hippel-Lindau tumor suppressor protein (pVHL) E3 ligase complex, leading to degradation via the ubiquitin–proteasome pathway [21]. Under hypoxic conditions, prolyl hydroxylase-mediated hydroxylation of HIF-1α is inhibited, causing the accumulation of HIF-1α, which in turn induces the high expression of downstream metabolism-related molecules such as GLUT1, HK2, and LDHA, as well as angiogenesis-related molecules such as vascular endothelial growth factor (VEGF) [20].

## Results

### High UHRF1 expression in OC is significantly associated with poor patient prognosis

**Fig. 1:**
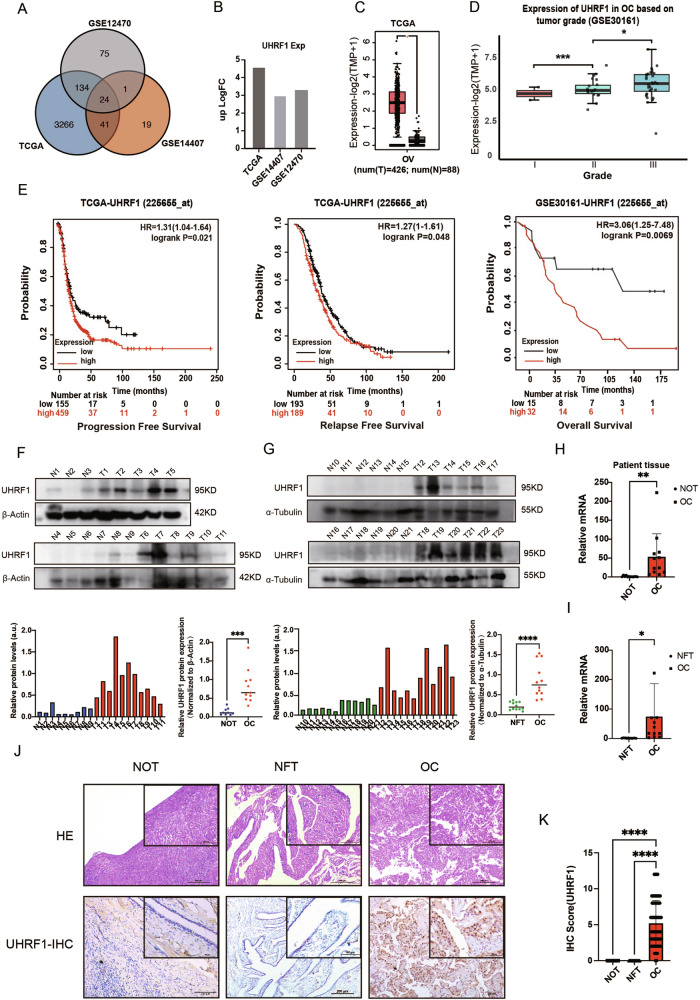
The validation and function prediction of UHRF1 in ovarian cancer (OC) patients from the TCGA-OV dataset. **A** Differential gene analysis was performed using the intersection of three OC datasets (TCGA-OV, GSE12470, and GSE14407). **B** UHRF1 expression across the datasets. **C** UHRF1 expression in TCGA-OV (normal vs. tumor samples). **D** Correlation between UHRF1 transcriptional levels and tumor grade in OC based on GSE30161 dataset analysis. **E** Kaplan–Meier analysis of UHRF1 expression and progression-free survival (PFS), relapse-free survival (RFS), and overall survival (OS) in OC patients. **F**, **G** Western blot (WB) analysis of UHRF1 expression in OC and normal tissues, N1–N9 represent normal ovarian tissue (NOT), while N10–N21 represent normal fallopian tube tissue (NFT). **H** qPCR analysis of UHRF1 transcription levels in OC and NOT. **I** qPCR analysis of UHRF1 transcription levels in OC and NFT. **J** Hematoxylin and eosin (HE) staining of OC, NOT, and NFT, and immunohistochemical (IHC) staining for UHRF1 expression. **K** Quantitative IHC analysis of UHRF1 levels in OC (*n* = 100), NOT (*n* = 50), and NFT (*n* = 50). *p < 0.05; **p < 0.01; ***p < 0.001; ****p < 0.0001.

To explore the risk factors affecting the occurrence and development of OC, differential analysis was performed on three OC datasets: TCGA-OV, GSE12470, and GSE14407. The upregulated genes in the OC group were intersected, resulting in 24 genes with elevated expression (Fig. 1A). Among these genes, UHRF1 was significantly increased in OC (Fig. 1B, C) and was associated with higher pathological grade (Fig. 1D). Furthermore, in both the TCGA dataset and our institutional OC cohort analyzed by immunohistochemical (IHC) staining, we observed a positive correlation between increasing pathological grade and elevated UHRF1 expression levels (Fig. S1A, B). Kaplan–Meier survival analysis revealed that patients with high UHRF1 expression had poor prognosis in OC (Fig. 1E). Furthermore, pan-cancer analysis revealed that UHRF1 is significantly overexpressed in 22 types of malignancies and is significantly associated with poor prognosis in various systemic tumors (Fig. S1C, D). These findings suggest that UHRF1 may be a potential risk factor for the occurrence and development of OC. To validate this hypothesis, we collected tumor samples from 24 OC patients, along with 12 NOT samples and 12 NFT samples from Xiangya Hospital. Western blotting (WB) and quantitative polymerase chain reaction (qPCR) validation demonstrated that UHRF1 was significantly overexpressed in the OC group compared with that in the control group (Fig. 1F–I). Additionally, the results of IHC results confirmed the high expression of UHRF1 in OC tissues (Fig. 1J, K). Follow-up was conducted on the patients with histological sections, and survival status and progression-free survival were used as outcome measures. Univariate and multivariate analyses indicated that older age, higher tumor grade, the presence of omental metastasis, and high UHRF1 expression are all independent risk factors for prognosis in OC patients (Table 1).

**Table 1:** Univariate and multivariate Cox regression of PFS analysis for OC patients.

Characteristics	Total (*N*)	Univariate analysis Hazard ratio (95% Cl)	*p* value	Multivariate analysis Hazard ratio (95% Cl)	*p* value
Age	100	1.089 (1.033–1.147)	0.002	1.092 (1.029–1.160)	0.004
Stage	100				
1	7	Reference		Reference	
2	5	1.344 (0.388–4653)	0.640	1.374 (0.387–4.875)	0.623
3	80	2.188 (0.984–4.865)	0.055	2.052 (0.793–5.309)	0.138
4	8	3.382 (1.021–11.197)	0.046	3.694 (0.974–14.010)	0.055
Lymph node metastasis	100				
_ −_	54	Reference		Reference	
^ +^	46	1.956 (0.889–4.302)	0.095	0.738 (0.304–1.794)	0.503
Omentum metastasis	100				
_ −_	37	Reference		Reference	
^ +^	63	2.964 (1.181–7.435)	0.021	1.509 (0.548–4.155)	0.426
UHRF1 Exp	100				
Low	22	Reference		Reference	
Middle	47	4.316 (0.513–36.277)	0.178	2.669 (0.306–23.314)	0.375
High	31	41.618 (5.172–334.902)	<0.001	29.542 (3.493–249.846)	0.002

Age was handled as a continuous variable; stage, lymph node metastasis, omentum metastasis, and UHRF1 Exp were treated as categorical variables.

### UHRF1 enhances the proliferation, migration, and invasion of OC cells

**Fig. 2:**
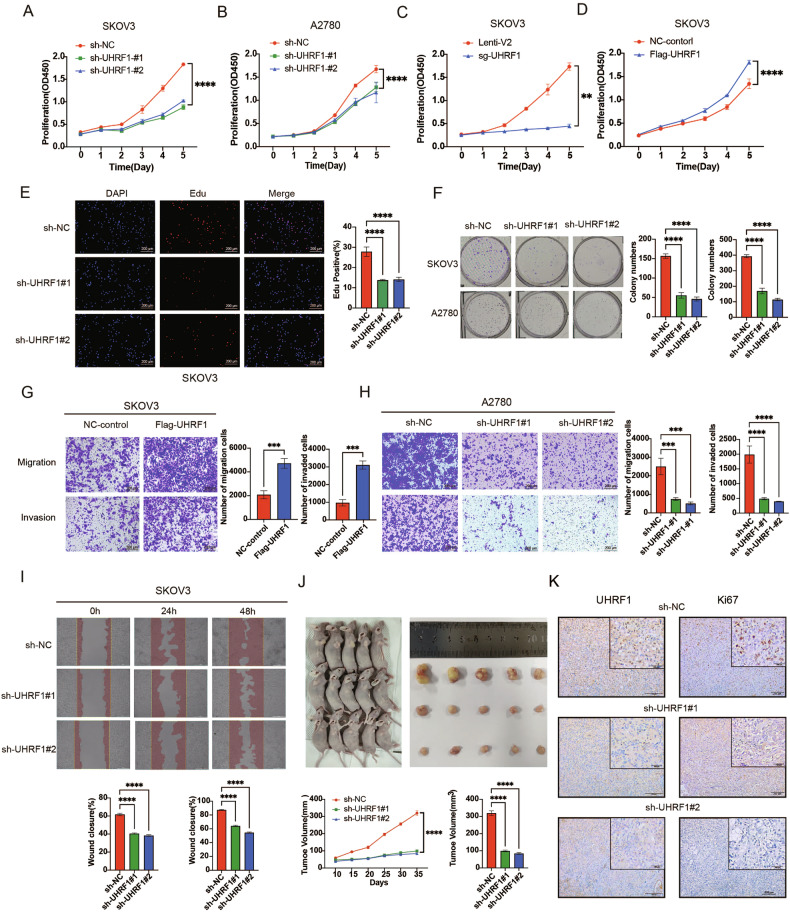
Overexpression of UHRF1 promotes proliferation, migration, and invasion of OC cells. **A**–**D** CCK8 assay to assess the effect of UHRF1-knockdown and UHRF1-overexpressed OC cell lines on the proliferation. **E** EDU cell proliferation assay showing the effect of UHRF1-knockdown OC cell lines. **F** Colony formation assay showing the effect of UHRF1-knockdown OC cell lines on colony-forming ability. **G**, **H** Transwell migration and invasion assays showing the effect of UHRF1-knockdown OC cell lines on migration and invasion abilities. **I** Wound healing assay showing UHRF1-knockdown SKOV3 cell lines migration. **J** Tumorigenicity assay in nude mice showing the effect of UHRF1-knockdown SKOV3 cell lines on tumor formation. **K** Ki67 staining of tumor tissues to assess the effect of UHRF1 knockdown on cell proliferation. **p < 0.01; ***p < 0.001; ****p < 0.0001.

To verify the impact of UHRF1 on the biological behavior of OC, we established UHRF1 knockdown and UHRF1-overexpression cell lines (SKOV3 and A2780) and validated them at the protein level (Fig. S2A, B). The results of the CCK-8, Edu proliferation, and colony formation assays indicated that knockdown of UHRF1 inhibited the proliferative capacity of OC cells, whereas overexpression of UHRF1 promoted cell proliferation (Figs. 2A–F and S2C–E). Transwell migration, invasion, and wound healing assays demonstrated that knockdown of UHRF1 weakened the migration and invasion abilities of OC cells, whereas UHRF1 overexpression enhanced these capabilities (Figs. 2G–I and S2F, G). In vivo tumorigenicity assays in nude mice revealed that UHRF1 knockdown significantly suppressed tumor growth in OC (Fig. 2J), and WB analysis of the tumor tissues confirmed the effectiveness of UHRF1 knockdown (Fig. S2H). IHC staining of tumor tissues from the tumor-bearing mice revealed that Ki67 expression was significantly reduced in the UHRF1 knockdown group (Fig. 2K), confirming that UHRF1 knockdown inhibited OC proliferation. These findings suggest that UHRF1 is a risk factor that promotes the occurrence and progression of OC, both in vitro and in vivo.

### UHRF1 regulates the HIF-1α pathway and positively modulates HIF-1α expression

**Fig. 3:**
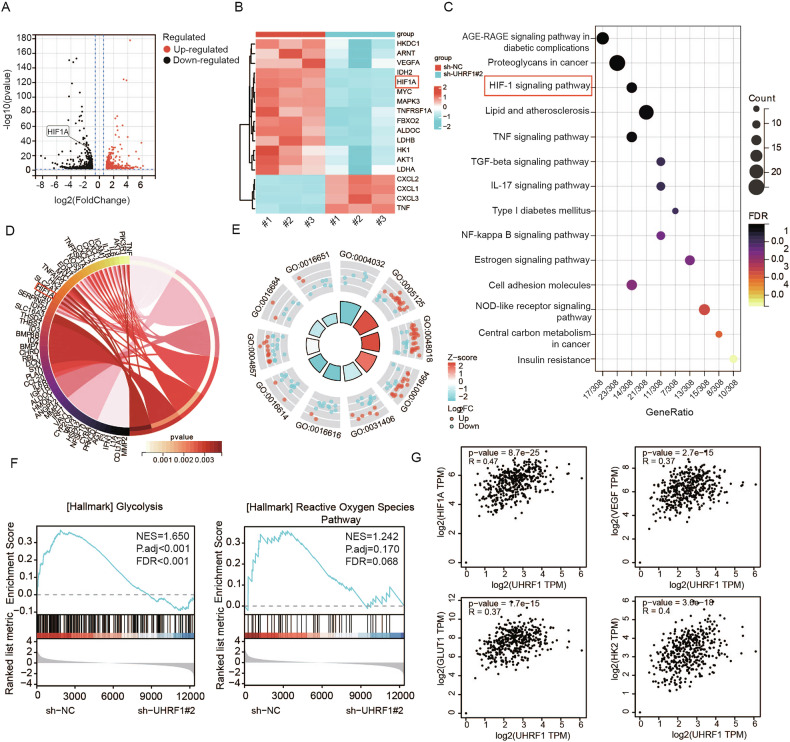
Transcriptome data suggest UHRF1 is involved in regulating the HIF-1 signaling pathway. **A** Volcano plot showing differential expression analysis between sh-NC SKOV3 cells and sh-UHRF1#2 SKOV3 cells, highlighting key potential downstream molecules. **B** Heatmap displaying the expression of HIF-1 signaling pathway genes between the knockdown and control groups. **C**, **D** KEGG pathway enrichment analysis of differentially expressed genes (DEGs), bubble chart shows the enriched pathways; circle plot displays the specific genes within the pathways. **E** GO-MF enrichment analysis of DEGs, the specific pathway names can be found in (Supplementary Table 3). **F** GSEA enrichment analysis of DEGs. **G** Correlation analysis of UHRF1 and HIF-1 signaling pathway genes using GEPIA2.

**Fig. 4:**
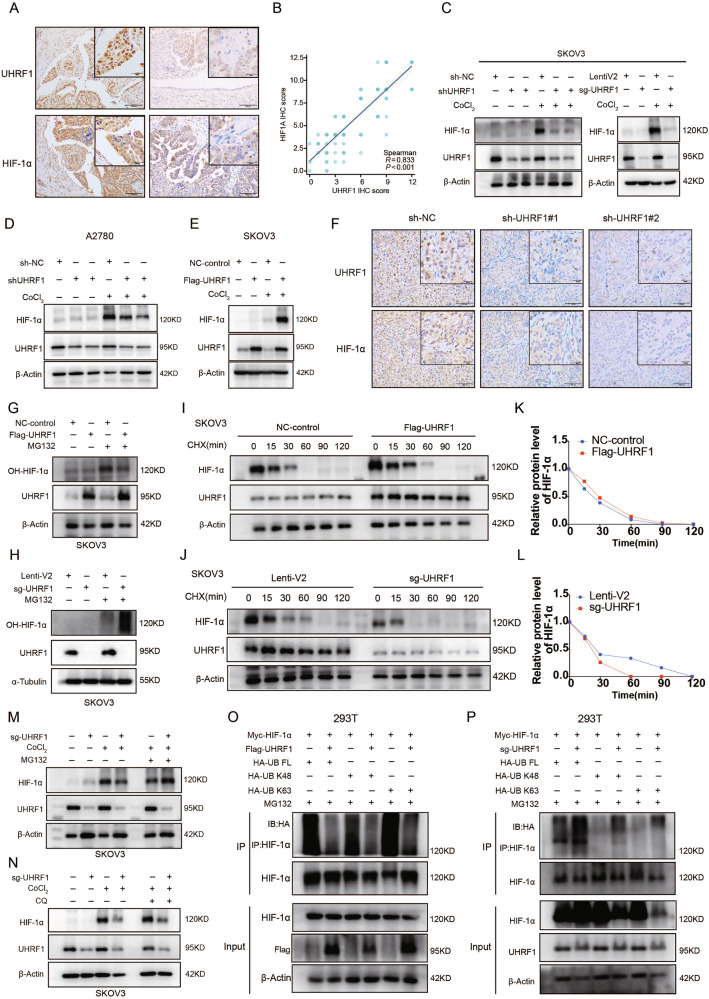
UHRF1 inhibits HIF-1α ubiquitin–proteasome degradation pathway. **A** Representative IHC images of UHRF1 and HIF-1α staining in OC patient tissue microarrays. **B** Correlation between UHRF1 and HIF-1α expression with corresponding *p* values indicated in the figure. **C**–**E** UHRF1-knockdown and UHRF1-overexpressed OC cell lines were seeded in a six-well plate, treated with CoCl_2_ for 24 h, and collected for WB analysis. **F** IHC staining of UHRF1 and HIF-1α in tumor-bearing mice tissue microarrays. **G**, **H** Cells were treated with 20 µM MG132 for 12 h, and collected for WB analysis to examine hydroxy-HIF-1α (OH-HIF-1α) expression in different groups. **I**–**L** Cells were treated with cycloheximide (CHX) and collected at different time points for WB analysis. **M** Cells were treated with 20 µM MG132 for 12 h and collected for WB analysis. **N** Cells were treated with 30 µM Chloroquine (CQ) for 12 h and collected for WB analysis. **O**, **P** 293T cells were transfected with the indicated plasmids, treated with MG132 for 12 h, and collected for WB analysis.

To further investigate the potential mechanisms by which UHRF1 promotes the malignant progression of OC, we performed transcriptome sequencing on UHRF1-knockdown stable SKOV3 cell lines. The results indicated that the expression of HIF-1α and its downstream key molecules was reduced in the UHRF1-knockdown group (Fig. 3A, B). Kyoto Encyclopedia of Genes and Genomes (KEGG) enrichment analysis revealed that UHRF1 was involved in regulating the HIF-1α signaling pathway, lipid metabolism-related pathways, and pathways associated with the AGE-RAGE signaling pathway, all of which are related to metabolic reprogramming (Fig. 3C, D). Gene ontology and gene set enrichment analyses revealed that UHRF1 also regulates biological behaviors such as tumor angiogenesis, glycolysis, and reactive oxygen species formation (Fig. 3E, F). Gene expression correlation results from GEPIA2 (http://gepia2.cancerpku.cn/) revealed a positive correlation between UHRF1 and the expression of HIF-1α and its downstream key molecules VEGF, GLUT1, and HK2 (Fig. 3G). Additionally, IHC results demonstrated that, in OC tissues, the group with high UHRF1 expression also presented relatively high levels of HIF-1α expression (Fig. 4A). Correlation analysis confirmed a statistically significant positive correlation between the expression of UHRF1 and that of HIF-1α (Fig. 4B). At the cellular level, the WB results indicated that under hypoxic conditions, UHRF1 knockdown led to a significant decrease in HIF-1α expression, wheras UHRF1 overexpression resulted in increased levels (Fig. 4C–E). Furthermore, IHC analysis of tumor tissues from tumor-bearing mice confirmed the positive correlation between UHRF1 and HIF-1α expression (Fig. 4F).

### UHRF1 inhibits HIF-1α degradation via the ubiquitin–proteasome pathway

Hydroxy-HIF-1α (OH-HIF-1α) serves as an intermediate product in the proteasomal degradation pathway of HIF-1α. The overexpression of UHRF1 led to a reduction in the expression levels of OH-HIF-1α, whereas UHRF1 knockdown resulted in an increase in its expression (Fig. 4G, H). CHX assay demonstrated that UHRF1 overexpression prolonged the half-life of HIF-1α, whereas UHRF1 knockdown shortened the half-life of HIF-1α (Fig. 4I–L). In UHRF1-knockdown SKOV3 cells treated with proteasome inhibitor MG132, UHRF1 deletion led to the downregulation of HIF-1α expression under hypoxic conditions, this effect was reversed by MG132 treatment (Figs. 4M and S3A). However, the lysosome inhibitor chloroquine did not exert any significant effect on HIF-1α expression (Figs. 4N and S3B).

To assess whether UHRF1 influences the ubiquitination level of HIF-1α, we co-transfected 293T cells with Flag-UHRF1, Myc-HIF-1α, and HA-tagged ubiquitin plasmids. After 48 h, cells were collected for WB analysis. The results indicated that overexpression of UHRF1 decreased the polyubiquitination of HIF-1α in 293 T cells (Fig. 4O), whereas knockdown of UHRF1 increased the ubiquitination of HIF-1α (Fig. 4P). These findings suggest that UHRF1 inhibits the degradation of HIF-1α through suppression of the ubiquitin-mediated proteasomal pathway.

PHD and pVHL are recognized as critical molecules in the classical proteasomal degradation pathway of HIF-1α. However, WB analysis showed no correlation between UHRF1 expression and the levels of PHD or pVHL (Fig. S3C, D). The specific mechanisms by which UHRF1 mediates the regulation of HIF-1α warrant further investigation.

### UHRF1 interacts with HIF-1α and significantly promotes its nuclear translocation

**Fig. 5:**
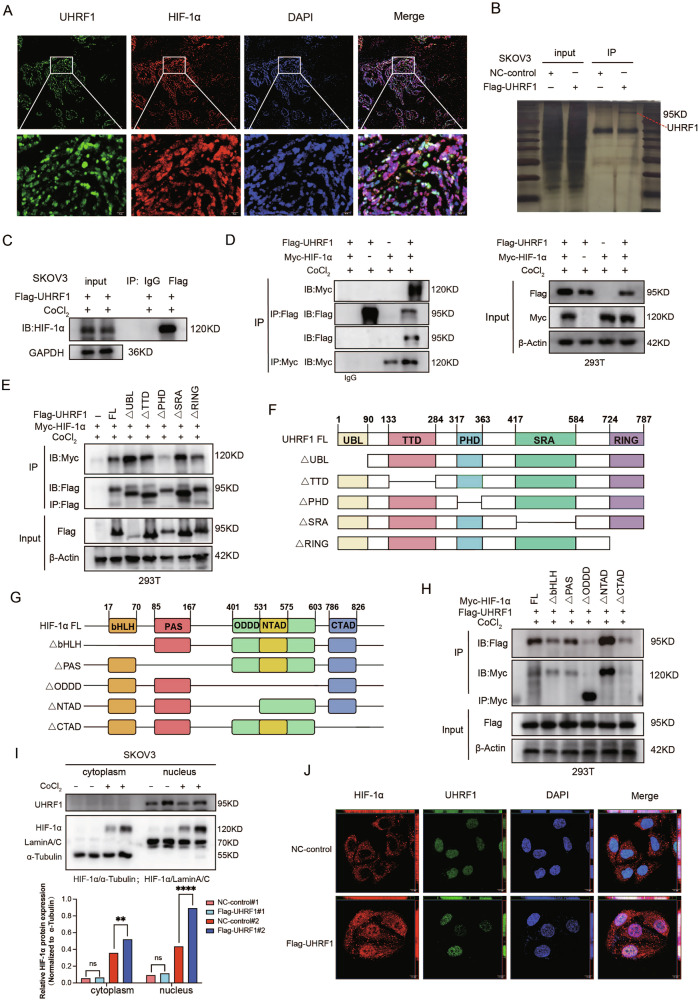
Interaction between UHRF1 and HIF-1α. **A** Tissue immunofluorescence (IF) confirmed the colocalization of UHRF1 and HIF-1α in tissues. **B** Silver staining of mass spectrometry samples. **C** Co-immunoprecipitation (co-IP) confirmed the interaction between UHRF1 and HIF-1α in SKOV3 cells. **D** Forward and reverse co-IP validated the interaction between UHRF1 and HIF-1α in 293T cells. **E** IB analysis of interaction domains between HIF-1α and UHRF1 using truncated plasmids co-transfected in 293T cells. **F** Truncated sequence map of UHRF1. **G** Truncated sequence map of HIF-1α. **H** WB analysis of interaction domains between UHRF1 and HIF-1α using truncated plasmids co-transfected in 293T cells. **I** WB analysis of UHRF1’s effect on the nuclear translocation of HIF-1α. **J** IF analysis of UHRF1’s effect on HIF-1α nuclear translocation.

Immunofluorescence (IF) results revealed extensive co-localization of UHRF1 and HIF-1α in OV tissues (Fig. 5A). To further investigate the mechanism by which UHRF1 inhibits HIF-1α degradation, we performed a pull-down assay using a Flag antibody in UHRF1-overepressed SKOV3 cells under hypoxic conditions, followed by systematic mass spectrometry analysis. The results demonstrated a direct interaction between UHRF1 and HIF-1α (Figs. 5B and S3E). Additionally, both endogenous and exogenous co-immunoprecipitation (co-IP) assays confirmed a strong interaction between UHRF1 and HIF-1α in 293T and SKOV3 cells (Fig. 5C, D).

To delineate the specific domains involved in their interaction, we constructed truncated forms of UHRF1 (ΔUBL, ΔTTD, ΔPHD, ΔSRA, and ΔRING) and HIF-1α (ΔbHLH, ΔPAS, ΔODDD, ΔNTAD, and ΔCTAD). Co-IP experiments revealed that the interaction between the truncated form of the ΔPHD of UHRF1 and HIF-1α was weakened, whereas the interaction between the truncated form of the ΔODDD of HIF-1α and UHRF1 was also diminished (Fig. 5E–H). These findings suggest that there is an interaction between UHRF1 and HIF-1α, which is mediated primarily by the UHRF1-PHD and HIF-1α-ODDD domains.

WB analysis confirmed that UHRF1 overexpression had a greater promoting effect on nuclear HIF-1α expression than on cytoplasmic HIF-1α expression (Fig. 5I). Similarly, the IF results demonstrated that under hypoxic conditions, UHRF1 overexpression significantly increased HIF-1α expression and facilitated its nuclear translocation, with a clear co-localization observed between the two proteins in the nucleus (Fig. 5J).

### UHRF1 mediates metabolic reprogramming in OC through HIF-1α

**Fig. 6:**
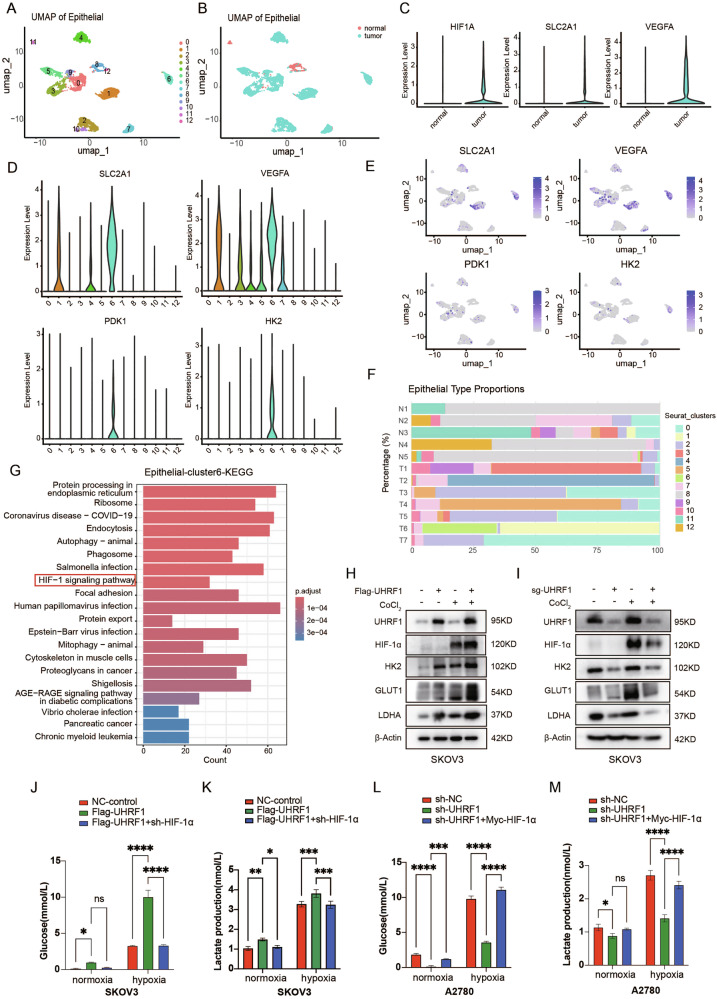
UHRF1 regulates metabolic reprogramming in OC cells via HIF-1α. **A** UMAP plot with clusters demarcated by colors demonstrating 12 distinct clusters based on gene expression differences for epithelial cells passing quality control. **B** The UMAP plot demarcated by colors showing the two groups of OC tumors (malignant) and nonmalignant ovarian tissues. **C** Expression of HIF signaling pathway genes in tumor and normal tissues. **D** Expression of HIF signaling pathway genes in each epithelial cell cluster. **E** UMAP plot color coded for the expression (blue to purple) of marker genes for HIF signaling pathway genes. **F** The proportion of epithelial cell types relative to the total epithelial cell count in each clinical patient. **G** KEGG pathway enrichment analysis of epithelial-cluster6 genes. **H**, **I** UHRF1-knockout and UHRF1-overexpressed SKOV3 cell lines were treated with CoCl₂ for 24 h, and WB analysis was performed to assess the expression levels of glycolysis-related proteins. **J**–**M** UHRF1-knockdown and UHRF1-overexpressed OC cell lines were cultured under normoxic and hypoxic conditions for 48 h, and glucose consumption and lactate production were measured. Data are presented as mean ± SD from three independent experiments. *p < 0.05; **p < 0.01; ***p < 0.001; ****p < 0.0001.

By analyzing the single-cell dataset GSE184880, we found significant differences in the cellular distribution between OC tumor tissues and normal control tissues. The normal tissues are predominantly composed of fibroblasts, while the tumor tissues are mainly composed of T cells and epithelial cells (Fig. S4A–D). The expression levels of SLC2A1 and VEGFA are higher in the tumor tissues compared to normal tissues (Fig. S4E). It is well known that OC primarily originates from epithelial cells, so we performed dimensionality reduction and clustering analysis on the epithelial cells (Fig. 6A, B). We examined the expression of genes in the HIF signaling pathway in these cells and found that, compared to the normal controls, epithelial cells derived from OC patients exhibit high expression of HIF signaling pathway genes (Fig. 6C). Further analysis identified a specific cluster of epithelial cells, designated as Cluster 6, which displayed high expression of HIF signaling pathway genes, including SLC2A1, VEGFA, PDK1, and HK2. This cluster was predominantly derived from Patient 6, whose clinical data indicated a higher clinical stage and a history of tumor recurrence (Figs. 6D–F and S4F). KEGG functional enrichment analysis demonstrated that Cluster 6 was closely associated with the HIF signaling pathway (Fig. 6G).

To determine whether HIF-1α is a critical mediator of UHRF1-promoted OC progression, we established stable cell lines of SKOV3 and A2780 cell lines with UHRF1 knockdown while simultaneously overexpressing HIF-1α, or conversely, overexpressing UHRF1 while knocking down HIF-1α. Changes in protein levels were validated using WB analysis (Fig. S5A, B). The results indicated that under CoCl₂-induced hypoxic conditions, UHRF1-overexpressed SKOV3 and A2780 cells presented increased expression of HIF-1α downstream metabolism-related molecules, including GLUT1, HK2, and LDHA. In contrast, UHRF1 knockdown resulted in reduced expression levels of these molecules (Fig. 6H, I).

Glucose uptake and lactate production assays further confirmed that UHRF1 overexpression increased the glucose uptake capacity of OC cells and increased lactate production, whereas UHRF1 knockdown inhibited these processes. Additionally, measurements of plasma glucose levels in tumor-bearing mice revealed that the plasma glucose concentration was greater in the UHRF1-knockdown group than in the control group, indicating that UHRF1 knockdown suppresses the ability of tumor cells to take up glucose (Figs. 6J–M and S5C, D)

### UHRF1 mediates tumor angiogenesis in OC through HIF-1α

Cell–cell communication analysis revealed the presence of epithelial–endothelial interactions predominantly mediated by VEGF–VEGFR signaling in OC tumors, which were absent in normal tissues (Fig. S6A, B). In addition, transcriptomic analysis revealed that in the UHRF1 knockdown group, the expression of several angiogenesis markers, such as CD34 and NRP1, as well as vascular smooth muscle cell differentiation markers like ANPEP and PDGFRL, was reduced (Fig. S6C).

**Fig. 7:**
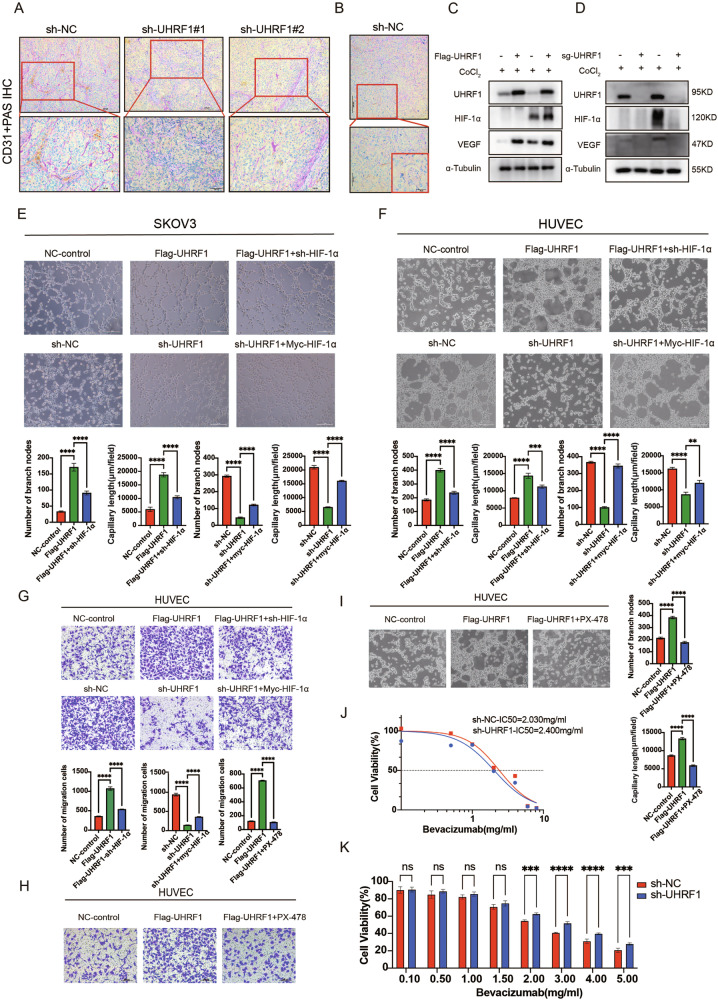
UHRF1 participates in the regulation of tumor angiogenesis and VM in OC via HIF-1α. **A**, **B** Periodic acid-Schiff (PAS) (pink) and CD31 (brown) double staining of tumor tissues from xenograft mice. PAS+/CD31+ tubular structures represent conventional blood vessels lined with CD31-positive endothelial cells. Adjacent PAS+/CD31− tubular structures suggest vasculogenic mimicry (VM). **C**, **D** WB analysis of VEGF expression levels in cells treated with CoCl₂ for 24 h. **E** Representative images of the tube formation assay in SKOV3 stable cell lines. **F** Representative images of the tube formation assay in HUVEC cells after 72 h of culture with conditioned medium from the stable SKOV3 cell line. **G** Images of the Transwell migration assay and tube formation assay in HUVEC cells after 72 hours of culture with conditioned medium from the stable SKOV3 cell line. **H**, **I** Images of the Transwell migration assay and tube formation assay in HUVEC cells after 72 h of culture with conditioned medium from the stable SKOV3 cell line, followed by treatment with the HIF-1α inhibitor PX-478. **J**, **K** IC50 analysis of Bevacizumab in UHRF1-knockdown A2780 cell lines. **p < 0.01; ***p < 0.001; ****p < 0.0001.

OC, characterized as a rapidly progressing and highly malignant solid tumor, requires an extensive vascular network to supply oxygen and nutrients. Co-staining for CD31 and PAS in tissue sections from OC patients revealed a substantial vascular network within the tumor tissue (Fig. S6D). Additionally, we unexpectedly observed scattered cavities surrounded by tumor cells within the OC tissue, which contained platelet components (Fig. S6E). This phenomenon is referred to as vascular mimicry (VM). HE staining of tumor tissue sections from tumor-bearing mice demonstrated a significant reduction in tumor vascular distribution in the UHRF1 knockdown group compared to the control group (Fig. S6F). Furthermore, the results of CD31 and Periodic acid-Schiff (PAS) co-staining also confirmed a decreased vascular distribution within the tumors of the UHRF1 knockdown group (Fig. 7A). In the control group, a VM phenomenon similar to that observed in human OC tissue was noted (Fig. 7B), which was absent in the UHRF1 knockdown group. WB results revealed that the overexpression of UHRF1 corresponded with an increase in VEGF expression, whereas the knockdown of UHRF1 resulted in decreased VEGF levels (Fig. 7C, D).

To investigate the roles of UHRF1 and HIF-1α in angiogenesis and VM, we performed VM analyses on human umbilical vein endothelial cells (HUVECs) and stable SKOV3 cells. The results indicated that the overexpression of UHRF1 promoted vascular formation; however, the knockdown of HIF-1α in UHRF1-overexpressed cells diminished this pro-angiogenic effect, and vice versa (Fig. 7E, F). Furthermore, overexpression of UHRF1 increased the migratory capacity of HUVECs, whereas knockdown of HIF-1α reduced HUVEC migration. Additionally, UHRF1 knockdown decreased the migration ability of HUVECs; however, this suppression was partially restored by the supplementation of HIF-1α. (Fig. 7G). Under conditions of UHRF1 overexpression, the addition of the HIF-1α inhibitor PX-478 suppressed the migratory capacity of HUVECs (Fig. 7H). Similarly, the results from the vascular formation experiments revealed that UHRF1 overexpression facilitated vascular formation, which was inhibited upon the addition of the HIF-1α inhibitor PX-478 (Fig. 7I). The CCK8 assay results demonstrated that in A2780 cells, the knockdown of UHRF1 led to an increased IC50 value for bevacizumab (Fig. 7J–K), suggesting that UHRF1 may serve as a potential target for bevacizumab.

### UHRF1 partially promotes the progression of OC via the HIF-1α pathway

**Fig. 8:**
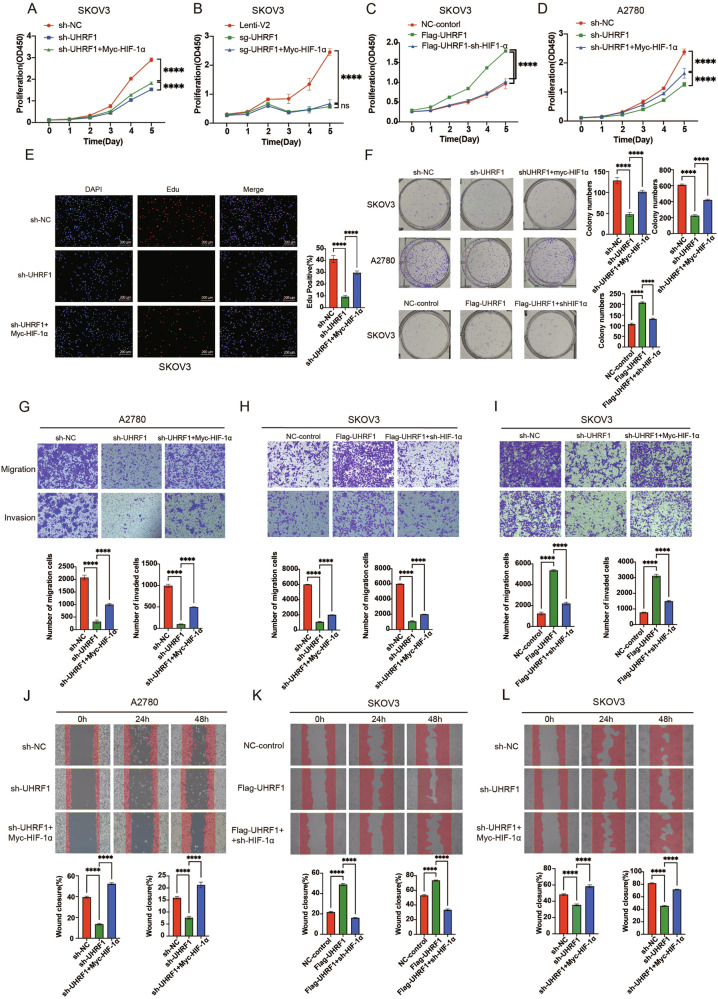
Knockdown of HIF-1α partially attenuates the promotion of malignant phenotypes in OC by UHRF1. **A**–**D** CCK8 assay to assess changes in proliferation capacity of UHRF1-knockdown and HIF-1α-overexpressed OC cell lines or UHRF1-overexpressed and HIF-1α-knockdown OC cell lines. **E** EDU cell proliferation assay to evaluate the proliferation capacity of double stable transfected cells. **F** Colony formation assay to examine the colony formation ability of double stable transfected cells. **G**–**I** Transwell migration and invasion assays to assess the migration and invasion capacities of double stable transfected cells. **J**–**L** Wound healing assays to evaluate the migration capacity of double stable transfected cells. ****p < 0.0001.

CCK8, Edu cell proliferation, and colony formation assays confirmed that the expression of HIF-1α partially restored the reduced proliferative capacity of OC cells resulting from UHRF1 knockdown. Additionally, HIF-1α knockdown also diminished the pro-proliferative effect of UHRF1 overexpression on OC cells (Figs. 8A–F and S7A, B). Transwell migration, invasion, and scratch assays demonstrated that HIF-1α expression partially reversed the diminished migratory and invasive capabilities of OC cells following UHRF1 knockdown. Similarly, HIF-1α knockdown attenuated the promoting effects of UHRF1 overexpression on the migration and invasion of OC cells (Fig. 8G–L).

## Discussion

As a highly malignant and easily metastatic solid tumor, OC poses a significant threat to women’s health due to its high recurrence rate and mortality [22]. In an effort to address this critical health challenge, numerous researchers have tirelessly investigated the mechanisms potentially involved in its pathogenesis, aiming to identify therapeutic targets [23**–**25]. Our study revealed that UHRF1 is overexpressed in OC and is associated with advanced pathological stages and poor prognosis, identifying it as a potential biomarker for OC. Transcriptome sequencing suggests a potential link between UHRF1 and the HIF-1 signaling pathway, which is crucial for the regulation of cell proliferation and metabolism. We found that UHRF1 inhibits the ubiquitin–proteasome degradation pathway of HIF-1α, leading to its accumulation and nuclear translocation, where it acts as a transcription factor to promote the expression of key molecules in glycolysis and angiogenesis pathways [19, 20, 26]. Knockdown of UHRF1 significantly suppressed the malignant phenotype of OC. This study is the first to demonstrate the role of UHRF1 in metabolic reprogramming and tumor angiogenesis in OC, offering new possibilities for therapeutic strategies and targeted drug development.

As a multifunctional protein, UHRF1 has been extensively demonstrated to play a critical role in tumorigenesis by regulating biological processes such as cell proliferation through its control of DNA methylation and DNA damage repair mechanisms [27**–**29]. However, the effects of UHRF1 on cellular metabolism and metastasis have been scarcely studied [15, 17]. Some studies suggest that UHRF1 acts as a gatekeeper of AMPK activity, regulating lipid and glucose metabolism at both cellular and animal levels [17]. Knockdown of UHRF1 has been shown to promote AMPK phosphorylation, inhibit HIF-1α expression, and consequently reduce glycolytic activity in tumor cells. Nevertheless, this study uncovers an alternative regulatory mechanism between UHRF1 and HIF-1α under hypoxic conditions, whereby UHRF1 enhances HIF-1α levels by inhibiting its degradation through the ubiquitin-proteasome pathway in OC.

It is well established that HIF-1α is highly unstable under normoxic conditions, with its primary degradation pathway being mediated by PHD and pVHL through the ubiquitin-proteasome system. Previous studies have indicated that certain molecules, such as PIN1, CDK1, or specific small interfering RNAs, can modulate HIF-1α degradation by influencing the levels of PHD and pVHL or their interaction with HIF-1α, thereby affecting the activation of the HIF-1 signaling pathway [30**–**32]. Moreover, reports suggest that under hypoxia, SUMO-specific protease 1 (SENP1) mediates the deSUMOylation of Elongin C, promoting the deubiquitination of HIF-1α by USP51 [33]. OH-HIF-1α is an intermediate in the HIF-1α degradation pathway. In this study, we observed that the ubiquitin-proteasome degradation of HIF-1α persists in OC cells under hypoxic conditions, as indicated by the accumulation of OH-HIF-1α upon the administration of the proteasome inhibitor MG132. Notably, UHRF1 expression was found to be inversely correlated with OH-HIF-1α levels, while no significant impact on PHD or pVHL expression was observed. Additionally, we discovered an interaction between UHRF1 and HIF-1α, with the PHD domain of UHRF1 and the ODDD domain of HIF-1α serving as the primary binding sites. The ODDD domain is crucial for HIF-1α degradation via the ubiquitin-proteasome pathway. Thus, we hypothesize that UHRF1 may modulate HIF-1α through post-translational modifications that interfere with PHD-mediated hydroxylation of HIF-1α, or alternatively, the binding of UHRF1 to HIF-1α may disrupt the interaction between HIF-1α and PHD, thereby affecting OH-HIF-1α levels and subsequent degradation. Further investigation is required to elucidate the precise mechanism.

Most solid tumors, such as liver cancer and OC, grow in environments characterized by hypoxia, low glucose levels, and acidity [8]. To adapt to the high demands for energy and oxygen necessary for rapid growth, tumor cells modify their nutrient uptake mechanisms and promote angiogenesis to enhance their self-sustenance capacity [6, 34]. For example, MYC through the activation or inhibition of specific enzymes, cooperates with HIF to activate genes in the glycolytic pathway, accelerating glucose transport and oxidation rates to meet the energy requirements of tumors [35, 36]. Additionally, substantial evidence has linked tumor dissemination to alterations in glucose metabolism. HIF-1α and its target enzyme PDK1 are upregulated in liver metastatic cells, promoting glycolysis [37]. Furthermore, activation of glycolysis has been shown to significantly enhance the metastasis of OC cells. SIK2 stimulates the Warburg effect in OC cells by activating the PI3K/AKT pathway, impacting epithelial–mesenchymal transition and promoting OC cell invasiveness [38].

In this study, we found that overexpression of UHRF1 significantly enhances the nuclear translocation of HIF-1α. Once translocated to the nucleus, HIF-1α dimerizes with HIF-1β to form a functional transcription factor, mediating the transcription of downstream key molecules [19]. Experimental results demonstrated that in UHRF1-overexpressed SKOV3 cell line, both glucose uptake and lactate production increased. Upon knockdown of HIF-1α, these changes were partially reversed, suggesting that UHRF1 participates in metabolic regulation in OC through the HIF-1 signaling pathway, thereby providing a novel therapeutic target for OC.

Similar to metabolic reprogramming, pathological angiogenesis plays a critical role in tumorigenesis and tumor progression [39]. Tumor growth is heavily reliant on angiogenesis, as every increase in tumor cell number is accompanied by a corresponding increase in new capillary formation [40]. VEGF, a key molecule in angiogenesis, is well recognized for its role in promoting blood vessel formation [41, 42]. During tumor growth, tumors establish their vascular networks through various mechanisms, including angiogenesis, vascular co-option, and vasculogenic mimicry [34]. CD31 and PAS co-staining revealed abundant vascular structures within OC tissues, including cavities formed by tumor cells containing platelet components, indicating the presence of vasculogenic mimicry.

Angiogenesis assays demonstrated that overexpression of UHRF1 enhances both angiogenesis and VM, while the inhibition or knockdown of HIF-1α suppresses these processes. It is well established that bevacizumab, a monoclonal antibody targeting VEGFA, was the first angiogenesis inhibitor approved for clinical use [43, 44]. Initially approved for metastatic colorectal cancer in combination with chemotherapy [45], bevacizumab’s indications have since expanded to include metastatic breast cancer, non-small cell lung cancer, glioblastoma, renal cell carcinoma, OC, and cervical cancer [46–51]. In this study we found that the UHRF1-knockdown stable cell line A2780 exhibited reduced sensitivity to bevacizumab compared to the control group, suggesting that UHRF1 may serve as a potential target for bevacizumab therapy.

In conclusion, we have identified UHRF1 as a critical oncogene involved in the development and progression of OC. UHRF1 regulates OC metabolic reprogramming and tumor angiogenesis by modulating the HIF-1 signaling pathway. Our findings suggest that UHRF1 influences the ubiquitin–proteasome degradation of HIF-1α by affecting its hydroxylation, although the precise mechanisms of this regulation require further investigation. The functional impact of UHRF1 on OC is significant; HIF-1α overexpression significantly but incompletely reversed UHRF1 deficiency-induced suppression of proliferation and migration. This partial rescue suggests UHRF1 orchestrates OC progression through synergistic or parallel HIF-1α-dependent and -independent mechanisms. Moreover, as the growth of UHRF1-knockout stable cell line SKOV3 was severely impaired, and even the exogenous introduction of HIF-1α had limited capacity to restore its malignant phenotype, underscoring UHRF1’s pivotal role in OC development.

Thus, a deeper exploration of UHRF1’s mechanisms in OC progression will hold considerable value for the future prevention, diagnosis, and treatment of OC.

## Methods and materials

### Cell lines and culture conditions

Based on previous OC research methodologies [22, 23], the SKOV3 and A2780 cell lines were selected as the subjects for this study to investigate OC-related mechanisms. The human OC cell line SKOV3 (CL-0215) was purchased from Wuhan Procell Life Science & Technology Co., Ltd., and A2780 (STCC10706P-1) was purchased from Wuhan Servicebio Technology Co., Ltd. The human embryonic kidney epithelial cell line 293T and the HUVECs were obtained from the National Health Commission Key Laboratory of Tumor Proteomics, Xiangya Hospital (Changsha, China). SKOV3 and 293T cells were cultured in DMEM (Meisen), A2780 cells were cultured in RPMI 1640 (Meisen), and HUVECs cells were cultured in ECM (Cat. #1001; ScienCell). All culture media were supplemented with 10% FBS (NEWAERUM Ltd) and 100 U/mL of penicillin and streptomycin (#SL6040, Coolaber). All cells were cultured in an environment of 37 °C with 5% CO_2_. When hypoxia induction was needed, the cells were cultured at 37 °C in 1% O_2_, and some hypoxia models were constructed by adding CoCl_2_ (20 mM, 24 h).

### Liposome transfection and lentiviral infection

Expression plasmids of UHRF1 and HIF-1α were constructed in the pCDNA3.1 vector for transient transfection, and in the PCDH-CMV vector for lentivirus infection (stable expression). The lentiviral vector PLKO.1 was used for the expression of shRNA in different cells. In addition, sg-UHRF1 was constructed using CRISPR/Cas9 technology. Truncated versions of UHRF1 and HIF-1α were constructed based on pCDNA-Flag-UHRF1 and pCDNA-Myc-HIF-1α using homologous recombination, with specific primer sequences shown in Supplementary Table 1. Plasmids were transfected using polyplus jetPRIME (#101000046, Polyplus), and cell extracts were collected 48 h later. PEI (#HY-K2014, MedChemExpress), psPAX, PMD2G, and the corresponding plasmids were co-transfected into 293T cells to produce lentiviral particles. Virus supernatants were collected at 48 and 72 h after transfection, and the virus supernatants were added to the SKOV3 and A2780 cells. After 72 h of infection, transfected cells were selected with puromycin or hygromycin B. UHRF1-knockdown cell lines were used primarily for functional experiments; UHRF1-knockout cell lines were used mainly for WB analysis.

### Real-time qPCR (RT-qPCR)

RNAiso Plus (#9108, Takara) was used to extract RNA from cells according to the manufacturer’s instructions. Complementary DNA was synthesized using the HiScript-Q Select RT SuperMix (#R133-01, Vazyme) for qPCR using a reverse transcription kit. qPCR was performed with the Taq Pro Universal SYBR qPCR Master Mix (#Q712-02/03, Vazyme) on an ABI 7500 instrument (Applied Biosystems). The endogenous reference genes used for RNA quantification were GAPDH and β-actin. The primers for the target genes are listed in Supplementary Table 1.

### Immunohistochemistry

A total of 100 cases of OC, 50 cases of NOT, and 50 cases of NFT were collected from the Department of Pathology at Xiangya Hospital. The OC pathological type is high-grade serous ovarian carcinoma. NOT and NFT were primarily obtained from normal ovarian tissues of patients who underwent bilateral adnexectomy for uterine fibroids or uterine prolapse. IHC staining was performed according to the protocol provided with the kit (#PV-9000, ZSGB-BIO). The primary antibodies used are detailed in Supplementary Table 2. Patients were followed up to analyze factors affecting prognosis (Table 1), and this study received approval from the Medical Ethics Committee of Xiangya Hospital.

### WB and co-IP assays

THE cells were lysed in RIPA buffer (#P0013K, Beyotime), followed by the addition of 5× SDS reducing sample buffer and incubation in a metal bath at 95 °C for 10 min. For co-IP, the cell lysates were incubated in IP lysis buffer for 30 min, and a total of 1000 µg of protein was prepared for subsequent experiments. Two micrograms of primary antibody was added to each sample, and 2 µg of the corresponding IgG antibody was added as a negative control. The protein sample was mixed with the antibody and rotated at 4 °C overnight. The next day, 20 µL of Protein A/G was added to the tube and rotated at 4 °C for 2 h. The beads were washed three times with cold PBS at 600 rpm per minute, the supernatant was discarded, 40 µL of 2× SDS reducing sample buffer was added, and the mixture was heated in a metal bath for 10 min. The proteins from each sample were separated using SDS-PAGE at the appropriate concentration and then transferred to a PVDF membrane. The samples were blocked with 5% BSA for 1 h and incubated with the primary antibody overnight at 4 °C. The samples were subsequently incubated with the corresponding secondary antibody for 1 h, and the immunoreactive bands were observed with hypersensitive ECL (#17046, Zenbio) chemiluminescence solution. The original full-length blot images can be found in the Supplementary Material.

### IF assay

Cells were seeded in confocal dishes at a density optimized to ensure the predominance of single-cell post-adhesion. After 24 h of cell adhesion, the cells were fixed with 4% paraformaldehyde for 30 min, permeabilized with 0.3% Triton X-100 for 20 min, and blocked with 5% BSA for 1 h. The primary antibody was added, and the mixture was incubated overnight at 4 °C. The primary antibody was washed off, and the samples were incubated with the fluorescent secondary antibody under light-protected conditions for 1 h. The cell nuclei were stained with DAPI, the coverslips were mounted, and the samples were observed and photographed under a fluorescence microscope.

### Cell viability assay

A total of 1000 cells were inoculated into a 96-well plate, and 90 µL of culture medium containing 10% FBS was added. At 0, 24, 48, 72, 96, and 120 h, 10 µL of Cell Counting Kit-8 (CCK8, #E-CK-A362, Elabscience) was added, and the cells and medium were mixed well. After incubation for 2 h, the OD at 450 nm was measured, 5000 cells were inoculated into a 96-well plate, and eight concentrations of bevacizumab (HY-P9906, MedChemExpress) were added the next day at 0.05, 0.1, 0.5, 1, 2, 3, 4, 5, and 6 mg/mL. After 48 h, the medium was changed, and 10 µL of CCK8 was added to each well. After incubation for 1 h, the optical density (OD) was measured at 450 nm.

### Transwell assays

For the migration assay, 3 × 10^4^ cells in 200 µL of serum-free medium were seeded into the upper chamber of a transwell insert, placed in a 24-well plate, and 800 µL of culture medium containing 10% FBS was added to the lower chamber. After incubation for 36 h, the bottom of the chamber was fixed with 4% paraformaldehyde and stained with crystal violet, and the number of cells migrating through the pores was counted under a microscope in three fields of view per well, followed by statistical analysis. The invasion assay was performed similarly to the migration assay, but with the addition of a basement membrane matrix gel (culture medium: matrix gel = 8:1) coated on the bottom of the chamber. The remaining steps were the same.

### Cell proliferation assay

A total of 5 × 10^4^ cells were seeded into a 24-well plate and cultured overnight until the cells returned to a normal morphology. EdU working solution (# K1076, APExBIO) was added according to the kit instructions. After staining was completed, a fluorescence detection device was used to assess cell proliferation. EdU-labeled DNA emits red fluorescence, while the nuclei stained with nuclear dye emit blue fluorescence. The cell proliferation status was evaluated by observing the number and intensity of fluorescent signals.

### Colony formation assay

A total of 1000 cells were seeded into a 6-well plate, and 2 mL of culture medium containing 10% FBS was added. The medium was changed every 3 days. After incubation for 15 days, the culture dish was washed with PBS, fixed with 4% paraformaldehyde, and stained with crystal violet. The number of colonies was counted via ImageJ, and statistical analysis was performed.

### Wound healing assay

The cells were seeded into a six-well plate at a density that allowed them to cover the bottom of the plate within 24 h. When the cells reached 100% confluence, a scratch wound was created with a 10 µL sterile pipette tip, and the cells were washed with PBS to remove floating cells. Two milliliters of serum-free medium was added, and the scratch conditions were recorded at 0, 24, and 48 h using an inverted microscope. The wound size was quantified using ImageJ software, and the wound healing rate was calculated and analyzed.

### Xenograft tumor

Fifteen 4-week-old female BALB/c nude mice were purchased from Hunan Sileke Jingda Experimental Animal Co., Ltd. and housed at the Laboratory Animal Center of Xiangya Hospital, Central South University. The mice were randomly divided into three groups: SKOV3-sh-NC, SKOV3-sh-UHRF1#1, and SKOV3-sh-UHRF1#2. After 1 week of acclimatization, each group of nude mice was subcutaneously injected with the corresponding stably transfected SKOV3 tumor cells (2 × 10^6^) in the axillary region. Two weeks later, the tumor size was measured every 2–3 days. The following formula was used to calculate the tumor volume: (*V*, volume; *L*, length; *W*, width). At the end of the experiment, the mice were euthanized humanely, and tumor tissues were collected for subsequent experiments. The animal experiment was approved by the Animal Ethics Committee of Xiangya Hospital, Central South University.

### Single-cell RNA statistical analysis

The single-cell RNA sequencing (scRNA-seq) data used in this study were obtained from the Gene Expression Omnibus (GEO) database under the accession number GSE184880. The dataset comprises five normal control tissues and seven OC tissues. Raw data from all samples were merged and subjected to rigorous quality control and preprocessing. Cells with fewer than 200 detected genes and genes expressed in fewer than 3 cells were filtered out. Additionally, cells with unique feature counts exceeding 4000 or falling below 200 were excluded. The percentage of mitochondrial gene content was calculated for each cell, and cells with mitochondrial gene proportions greater than 40% were removed to ensure data quality. The remaining data were normalized using the Seurat package (version 3.1.4, https://satijalab.org/seurat/). Normalization and scaling were performed based on unique molecular identifier (UMI) counts and mitochondrial gene percentages for each sample. The scaled data were used to identify the top 2000 highly variable genes, which were subsequently utilized for principal component analysis (PCA). The first 10 principal components (PCs) were selected for downstream analysis. Uniform Manifold Approximation and Projection (UMAP) was employed for dimensionality reduction, and batch effects were corrected using the Harmony algorithm integrated within the Seurat package. Cell clustering was performed by constructing a k-nearest neighbor (KNN) graph and applying the Louvain algorithm for community detection. Cell clusters were annotated based on the expression of lineage-specific markers. Functional enrichment analysis of specific gene sets was conducted using the KEGG pathway database. Cell-cell communication patterns were explored using the CellChat package. Gene expression patterns were visualized using Seurat’s built-in functions, including FeaturePlot (UMAP), VlnPlot, and DotPlot.

### Glucose uptake and lactic acid production assay

The cells were seeded into a six-well plate and cultured under hypoxic conditions. The cells were lysed with 100 µL of RIPA lysis buffer after they had grown to confluence. After complete lysis, the mixture was centrifuged, and the supernatant was collected. The instructions for the glucose detection kit (O-toluidine method) (#S0201S, Beyotime) were followed for subsequent steps. After the reaction was complete, the optical density (OD) was measured at 630 nm. The standard curve was used to determine the glucose concentration of each group. Cells were seeded into a 10-cm dish and counted after they had grown to confluence. The instructions for the L-lactic acid assay kit (#A019-2-2, Nanjing Jiancheng) were followed for subsequent steps. After the reaction was complete, the OD was measured at 546 nm. The lactic acid concentration of each group was calculated, and statistical analysis was performed.

### Angiogenesis assay

Matrigel matrix (#356234, Corning) was used to detect angiogenesis. First, a 24-well plate was coated with Matrigel matrix (0.2 mL/well) and solidified at 37 °C for 1 hour. The stable cell line SKOV3 and the cell line HUVEC were cultured in conditioned medium, digested with trypsin, and resuspended at 10 × 10^4^ cells/mL in endothelial cell-conditioned medium and conditioned medium, respectively. The samples were seeded in 24-well plates coated with matrix gel and incubated at 37 °C for 1–2 h. Tube formation was observed, and the samples were examined under a microscope. Imaging analysis was performed with ImageJ software, and statistical analysis was performed.

### Reproducibility and statistics

All experiments, except for experiments involving nude mice, were repeated at least three times. The data were presented as the means ± SDs of at least three independent experiments. Two-tailed unpaired Student’s *t* tests were used for the intergroup comparisons. One-way analysis of variance was used to analyze the differences among three or more groups of samples. The Kaplan–Meier method was chosen to assess patients’ survival. *p < 0.05; **p < 0.01; ***p < 0.001; and ****p < 0.0001.

#### Ethics statement

The studies involving human participants were reviewed and approved by the ethics committee of the Xiangya Hospital, Central South University, China (202406122). All animal experiments were approved by the Ethics Committee of the Laboratory Animal Center, Central South University (202410170). All methods were performed in accordance with the relevant guidelines and regulations.

## Supplementary information

Supplementary materials

Original western blots

Supplementary materials—Publication License

## Data Availability

The data that support the findings of this study are available from the corresponding author upon reasonable request.
