# Supplementary material for: UHRF1-mediated HIF-1α stabilization promotes ovarian cancer through metabolic reprogramming and angiogenesis
Source: Cell Death Dis. 2025 Oct 24;16(1):751. doi: 10.1038/s41419-025-08033-w (PMC12552473; doi:10.1038/s41419-025-08033-w)
Supplement: Supplementary file 1 — Supplementary materials [file 41419_2025_8033_MOESM1_ESM.docx]

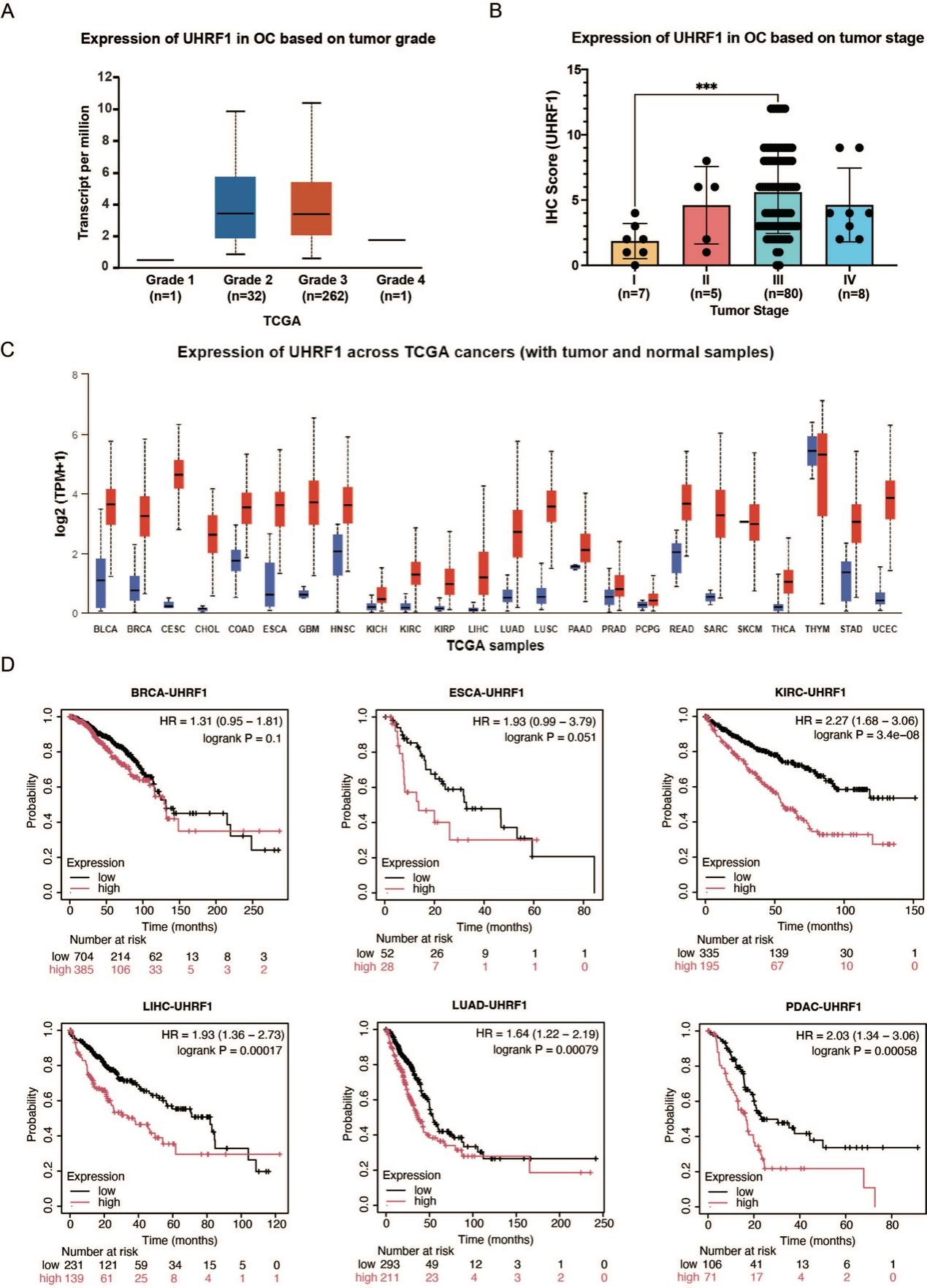


**Fig.S1: UHRF1 is overexpressed in various tumors and is associated with poor prognosis.** (A) Correlation between UHRF1 transcriptional levels and tumor grades in OC using the UALCAN (https://ualcan.path.uab.edu/) database; (B) The correlation between UHRF1 expression levels and tumor grade based on IHC score analysis in OC patients ;(C)Expression analysis of UHRF1 across pan-cancer using the UALCAN（https://ualcan.path.uab.edu/）database; (D) Prognostic analysis of UHRF1 in multiple tumors（Breast Cancer (BRCA), Esophageal Cancer (ESCA), Kidney Renal Clear Cell Carcinoma (KIRC), Liver Hepatocellular Carcinoma (LIHC), Lung Adenocarcinoma (LUAD), Pancreatic Ductal Adenocarcinoma (PDAC)）.


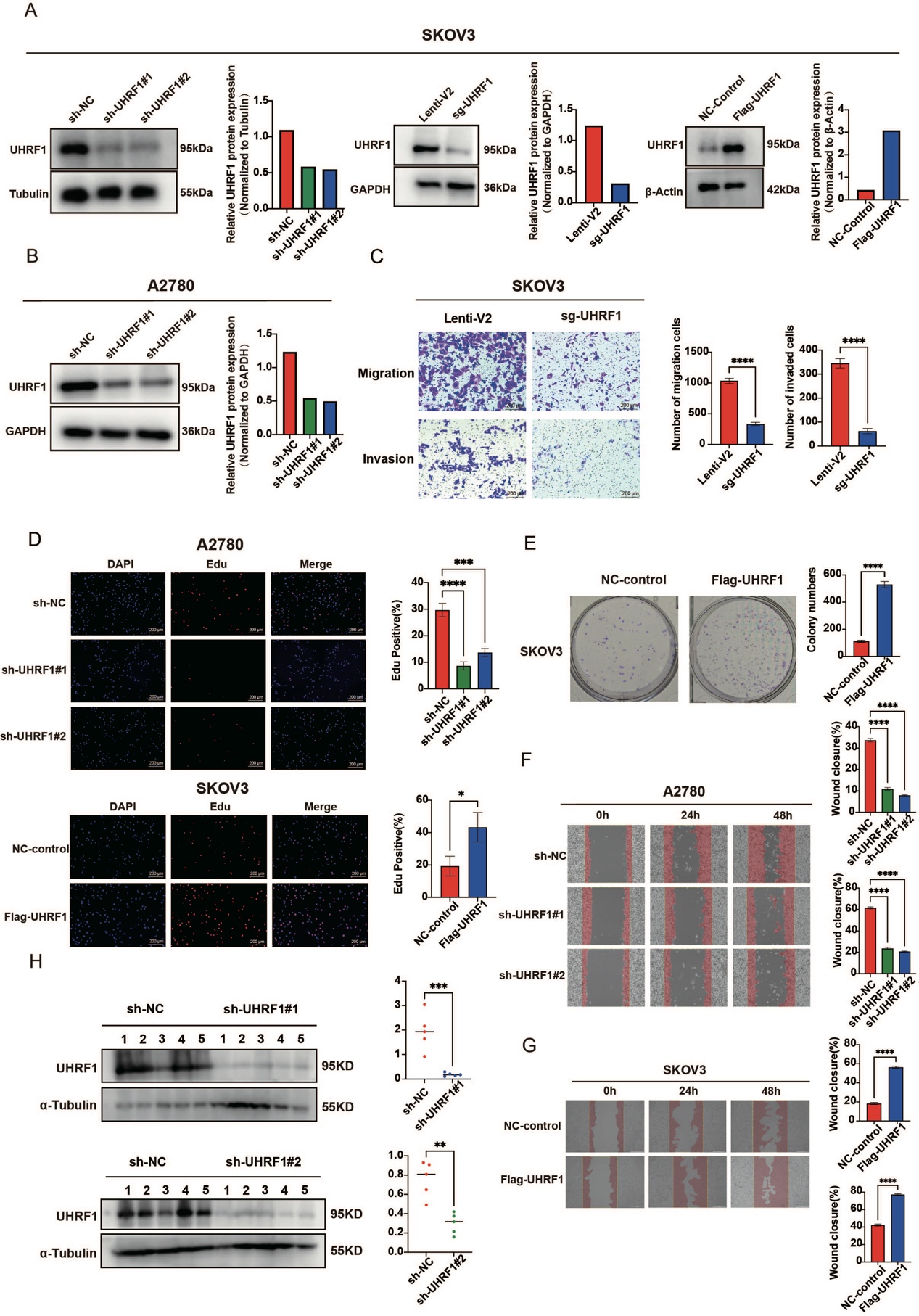


**Fig. S2: Knockdown of UHRF1 inhibits proliferation, migration, and invasion of OC cells.** (A, B) UHRF1-knockdown/knockout and UHRF1-overexpressed in OC cells, cells were collected to WB analysis and grayscale analysis; (C) Transwell migration and invasion assays showing the effect of UHRF1-knockout SKOV3 cells on migration and invasion abilities; (D) EDU cell proliferation assay showing the effect of UHRF1-knockdown and UHRF1-overexpressed SKOV3 cells on proliferation; (E) Colony formation assay showing the effect of UHRF1-overexpressed SKOV3 cells on colony-forming ability; (F, G) Wound healing assay showing the effect of UHRF1-knockdown A2780 cells and UHRF1-overexpressed SKOV3 cells on migration; (H) WB analysis of tumor tissues from xenograft mice. *p < 0.05; **p < 0.01; ***p < 0.001; ****p < 0.0001.


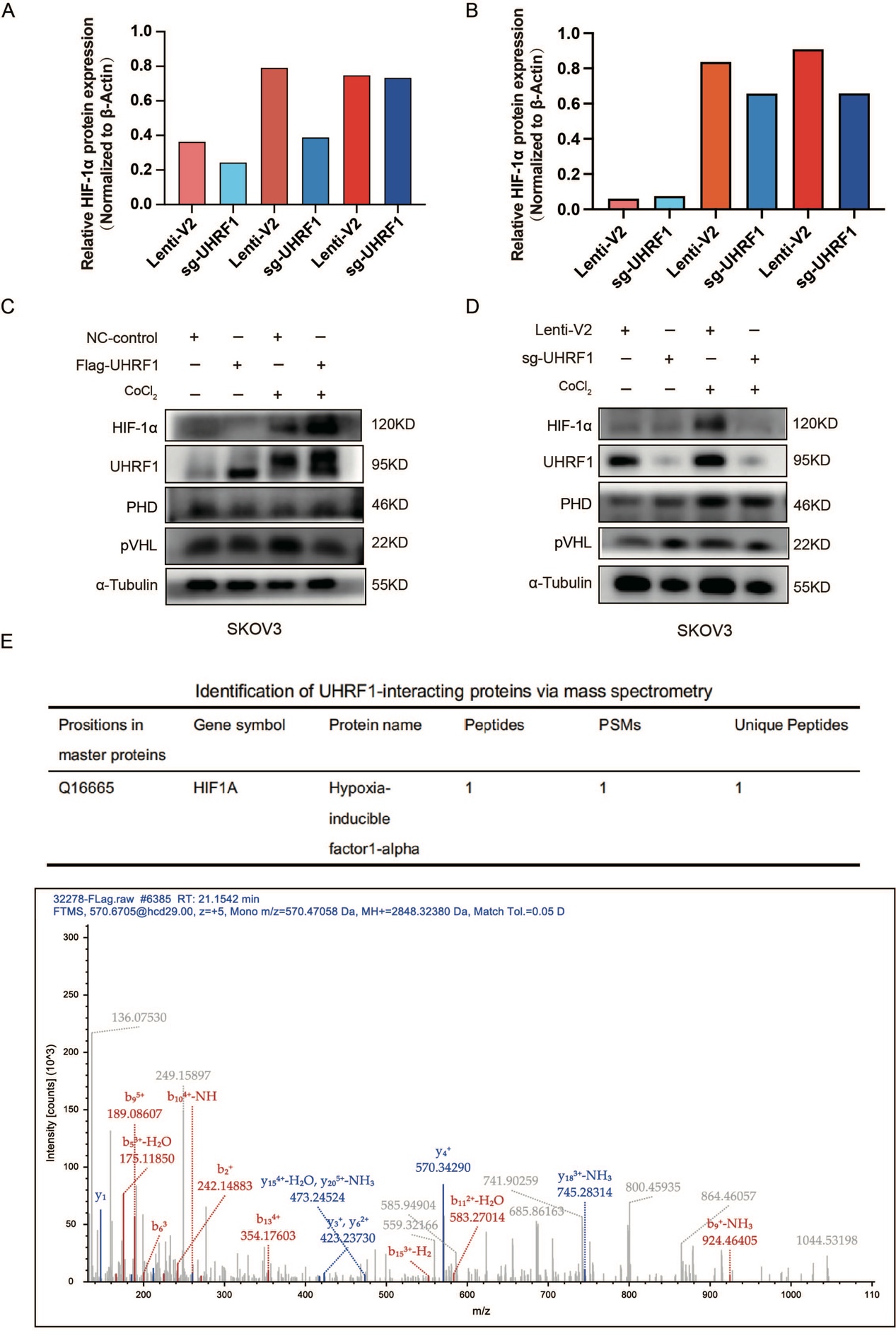


**Fig. S3: UHRF1 does not affect the expression of PHD and pVHL.** (A) Cells were treated with 20 µM MG132 for 12 h, and collected for grayscale analysis; (B) Cells were treated with 20 µM MG132 for 12 h, and collected for grayscale analysis; (C, D) UHRF1-overexpressed SKOV3 or UHRF1-knockout SKOV3 cells were treated with CoCl₂ for 24 h, and cells were collected for WB analysis; (E) Mass spectrometry assay identified proteins potentially interacting with UHRF1. Analysis results identified the interaction between UHRF1 and HIF-1α in SKOV3 cells.


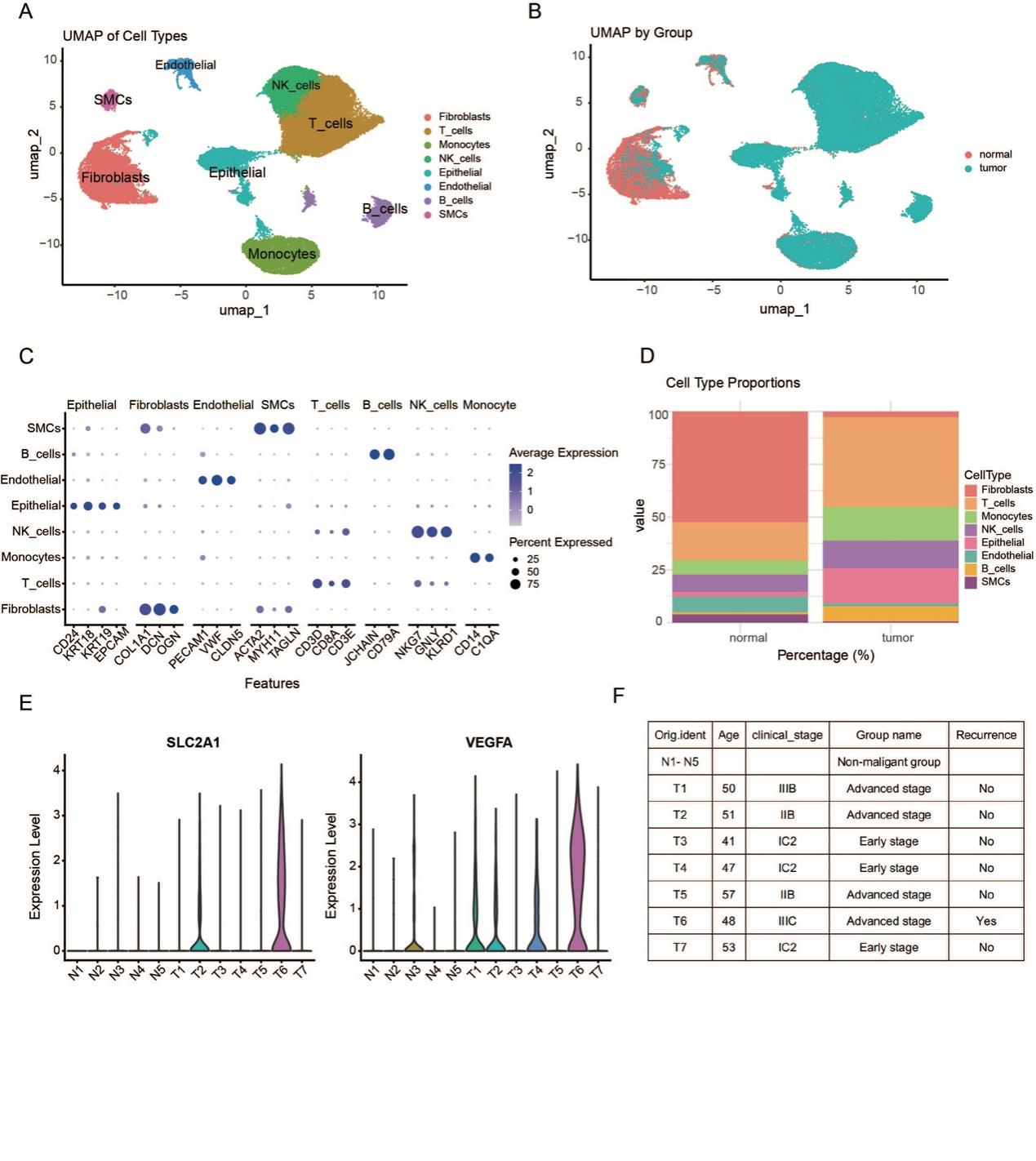


**Fig. S4: Diverse cell types in OC and nonmalignant ovarian tissues delineated by single-cell transcriptomic analysis.** (A) The UMAP plot demonstrates the main cell types in OC and control ovarian tissues; (B) The UMAP plot demarcated by colors showing the two groups of OC tumors and nonmalignant ovarian tissues; (C) Dot plots showing the expression levels of specific marker genes in each cell type. The size of dots indicates the proportion of cells expressing the particular marker gene. The spectrum of color represents the mean expression levels of the marker genes; (D) The proportion of each cell type relative to the total cell count in OC tumor and normal tissues; (E) The expression of SLC21A and VEGFA in each patient; (F) Clinical information of each patient.


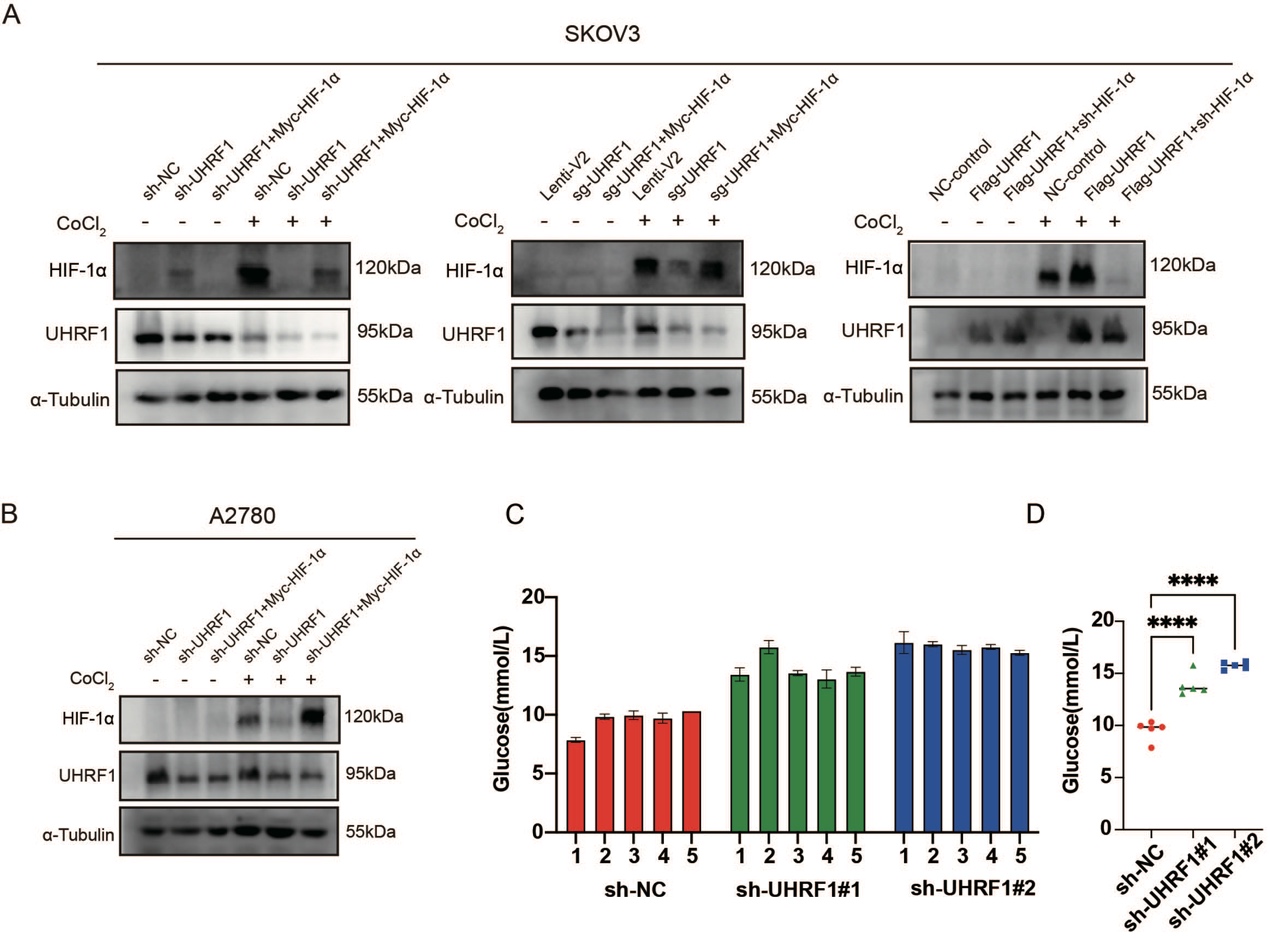


**Fig. S5: Construction of a bistable transgenic cell line.** (A-B) Generation of stable transfected cell lines, and WB analysis and grayscale analysis of the expression levels of relevant proteins; (C-D) Plasma glucose levels were measured from tumor-bearing mice and analyzed statistically.


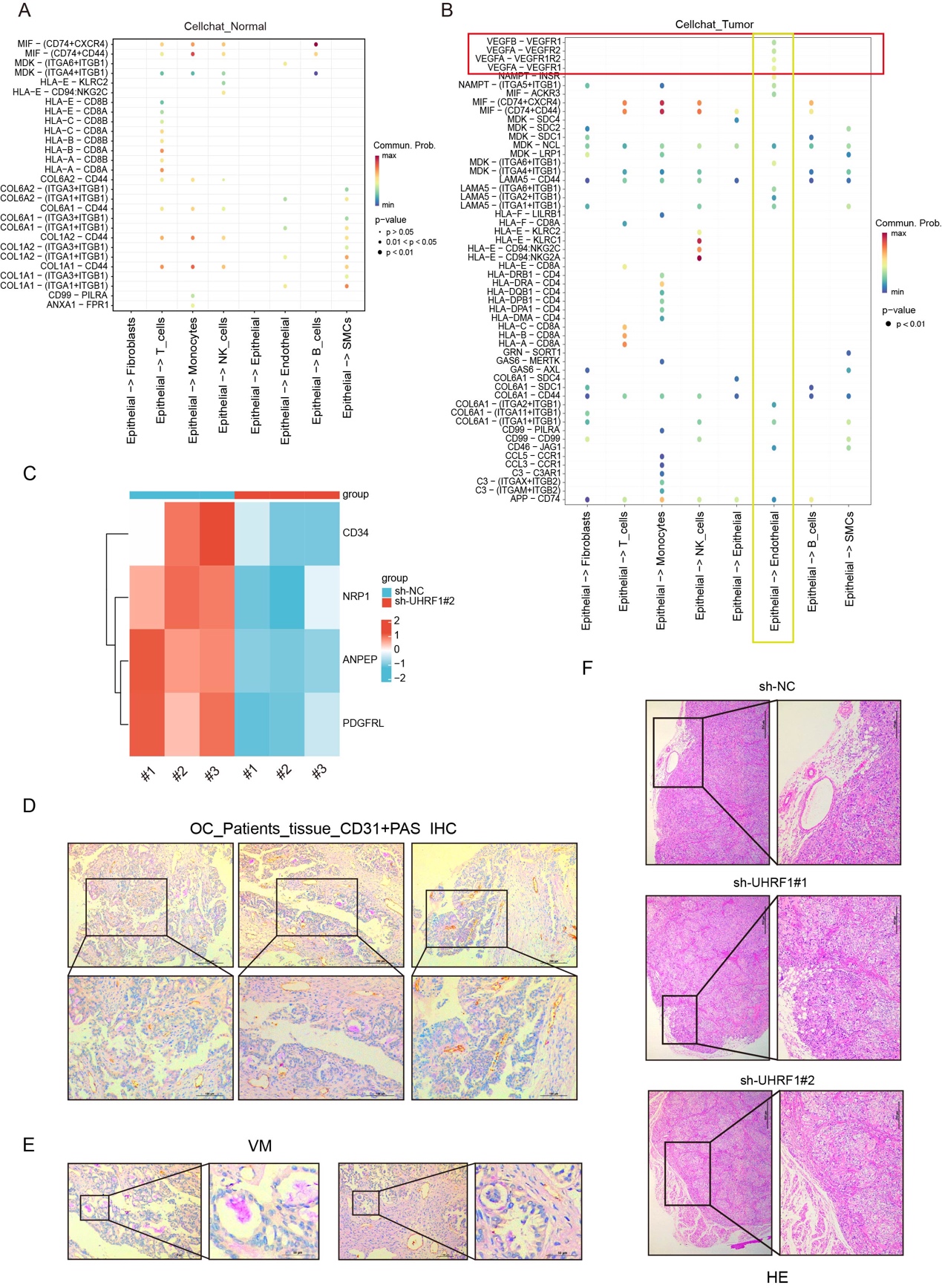


**Fig. S6: UHRF1 is involved in the regulation of tumor angiogenesis.** (A, B) Analysis of cell-cell communication between tumor and normal tissues; (C) Heatmap from transcriptomic analysis shows that knockdown of UHRF1 leads to a decrease in the expression of some angiogenesis-related genes; (D-E) CD31 and PAS co-staining of tumor tissues from OC patients indicates the presence of a large number of blood vessels and VM within the tumors; (F) HE staining of mouse tumor tissues.


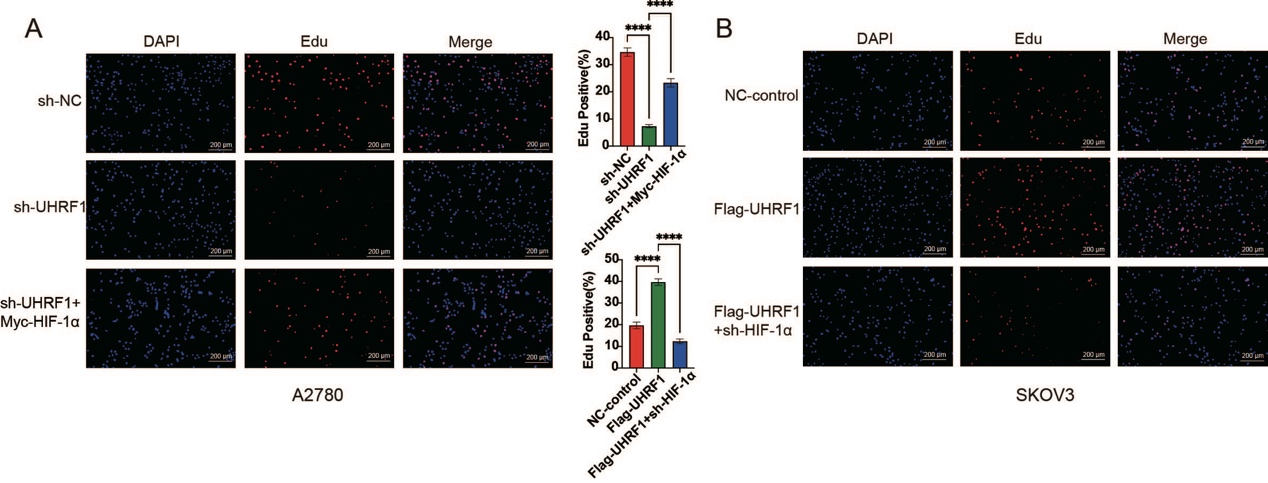


**Fig. S7:Knockdown of HIF-1α partially attenuates the promotion of malignant phenotypes in OC by UHRF1.** (A, B) EDU cell proliferation assay to evaluate the proliferation capacity of double stable transfected cells.

**Supplementary Table1**：Association of UHRF1 expression with clinicopathological features in OC patients

| Characteristics |  | UHRF1 expression | | χ² | P-value |
| --- | --- | --- | --- | --- | --- |
|  | （N） | Low（-、+）  （N=53） | High（++、+++）  （N=47） |  |  |
| Age, n (%) |  |  |  | 0.542 | 0.462 |
| < 50 | 40 | 23（57.5%） | 17（42.5%） |  |  |
| ≥ 50 | 60 | 30（50.0%） | 30（50.0%） |  |  |
| Stage, n (%) |  |  |  | 8.989 | **0.029^*^** |
| I | 7 | 7（100%） | 0（0.00%） |  |  |
| II | 6 | 3（50.0%） | 3（50.0%） |  |  |
| III | 79 | 37（46.8%） | 42（53.2%） |  |  |
| IV | 8 | 6（75.0%） | 2（25.0%） |  |  |
| Lymph node metastasis, n (%) |  |  |  | 0.915 | 0.339 |
| - | 54 | 31（57.4%） | 23（42.6%） |  |  |
| + | 46 | 22（47.8%） | 24（52.2%） |  |  |
| Omentum metastasis, n (%) |  |  |  | 7.032 | **0.008^*^** |
| - | 37 | 26（70.3%） | 11（29.7%） |  |  |
| + | 63 | 27（42.9%） | 36（57.1%） |  |  |

Tumors with low UHRF1 expression (-/+) showed IHC scores of 0-4, while those with high expression (++/+++) had scores of 6-12.

**Supplementary Table2:**

| Truncated Primer | |
| --- | --- |
| UHRF1-UBL F | GAATTCGCCACCATGTCCGACACCGACT |
| UHRF1-UBL R | CATGGTGGCGAATTCGCTAGCTCTAG |
| UHRF1-TTD F | AGCCGATGAGGACATGGGGAGCCCATGGTT |
| UHRF1-TTD R | CATGTCCTCATCGGCTGGCCCTGCTGT |
| UHRF1-PHD F | ACTGCAAGGACGACGTGGCCAGCGAGGT |
| UHRF1-PHD R | CACGTCGTCCTTGCAGTGCTTGCAGGAC |
| UHRF1-SRA F | ATGTGGCGGTTCCGAGTCCCTGGCCCTT |
| UHRF1-SRA R | GACTCGGAACCGCCACATGGTGCCCA |
| UHRF1-RING F | GAGGAGACGTTCCAGGGCTACGGCAAT |
| UHRF1-RING R | CTGGAACGTCTCCTCCACTTTACTCAGG |
| HIF-bHLH F | AAGATAAGTTCTGAAAAACTTCTGGATGCTGGTG |
| HIF-bHLH R | TTCAGAACTTATCTTTTTCTTGTCGTTCGCGCCG |
| HIF-PAS F | GGATATTGAAGATGACATGAAAGAACAAAACAC |
| HIF-PAS R | CATGTCATCTTCAATATCCAAATCACCAGCATCC |
| HIF-ODDD F | CTGATGCTTTAACTTTGCTGCAGACTCAAATAC |
| HIF-ODDD R | CAGCAAAGTTAAAGCATCAGGTTCCTTCT |
| HIF-NTAD F | GGATAGTGATATGGTCAATGAATCCTTCGATCAG |
| HIF-NTAD R | TTCATTGACCATATCACTATCCACATAAAAAC |
| HIF-CTAD F | TGTAGACTGCTGGGGCAAAACGAACAAAAA |
| HIF-CTAD R | TTGCCCCAGCAGTCTACATGCTAAATCAG |
| qPCR Primer | |
| GAPDH F | CAAGGCTGTGGGCAAGGTCATC |
| GSPDA R | GTGTCGCTGTTGAAGTCAGAGGAG |
| ACTIN F | CTTCGCGGGCGACGAT |
| ACTIN R | CCACATAGGAATCCTTCT |
| UHRF1 F | CACAACGTGTGCAAGGACTG |
| UHRF1 R | TTTCAGCAAAACGCCTGTCG |
| HIF1A F | GATCACCCTCTTCGTCGCTT |
| HIF1A R | CCTCCATGGTGAATCGGTCC |
| GLUT1 F | GGTTGTGCCATACTCATGACC |
| GLUT1 R | GAGATAGGACATCCAGGGTAGC |
| HK2 F | CCCGGGAAAGCAACTGTTTG |
| HK2 R | ACCGGTGTTGAGAAGCTCTG |
| LDHA F | CGTCAGCAAGAGGGAGAAAG |
| LDHA R | GCCACGTAGGTCAAGATATCC |
| VEGF F | GGGAAAGGGGCAAAAACGAA |
| VEGF R | GGAGGCTCCAGGGCATTAGA |

Truncated Primer was used for plasmid design; qPCR Primer was used for quantitative analysis.

**Supplementary Table3:**

| Antibolidies | | | | | |  |
| --- | --- | --- | --- | --- | --- | --- |
| Name | Vendor | | Cat# | WB | IHC | Validation |
| UHRF1 | Proteintech | | #21402-1-AP | 1:1000 | 1:200 | 1. WB validation using human and murine tissues 2. WB validation at the cellular level 3. IHC staining of human and murine tissues |
| HIF-1α | Proteintech | | #20960-1-AP | 1:1000 | 1:200 |  |
| GAPDH | Proteintech | | #60004-1-Ig | 1:5000 | - | Loading control protein |
| β-actin | Proteintech | | #66009-1-Ig | 1:5000 | - |  |
| α-tubulin | Proteintech | | #66009-1-Ig | 1:5000 | - |  |
| GLUT1 | ZEN-BIOSCIENCE | | #R380464 | 1:1000 | - | Pathway proteins validated by WB |
| HK2 | Proteintech | | #22029-1-AP | 1:1000 | - |  |
| LDHA | Cell Signaling Technology | | #3582T | 1:1000 | - |  |
| VEGF | Proteintech | | #19003-1-AP | 1:1000 | - |  |
| Hydroxy-HIF-1α | Cell Signaling Technology | | #3434 | 1:1000 | - |  |
| PHD | Proteintech | | # 19886-1-AP | 1:1000 | - |  |
| pVHL | Proteintech | | #24756-1-AP | 1:1000 | - |  |
| DYKDDDDK-tag | Proteintech | | #20543-1-AP; #66008-4-Ig | 1:1000 | - | 1. Tag protein 2. Used for enrichment in IP assays and detection by WB |
| MYC-tag | Proteintech | | #16286-1-AP; #60003-2-Ig | 1:1000 |  |  |
| HA-tag | Abmart | | #M20003 | 1:1000 |  |  |
| Ki67 | Abmart | | #TW0001 | - | 1:100 | IHC staining of mouse tissues |
| CD31 | Abmart | | #TA6191 | - | 1:100 | IHC staining of human and mouse tissues |
| Reagents | | | | | | |
| CoCl2 | | #102533，Merck KGaA | | | | |
| MG132 | | #S2619，Selleck，China | | | | |
| CHX | | #S7418，Selleck，China | | | | |
| PX-478 | | #S7612，Selleck，China | | | | |

Detailed information on antibody suppliers, catalog numbers, and applications.

**Supplementary Table4:**

**Enriched pathway**

| GO:0016651 | oxidoreductase activity, acting on NAD(P)H |
| --- | --- |
| GO:0004032 | alditol:NADP+ 1-oxidoreductase activity |
| GO:0005125 | cytokine activity |
| GO:0048018 | receptor ligand activity |
| GO:0001664 | G protein-coupled receptor binding |
| GO:0031406 | carboxylic acid binding |
| GO:0016616 | oxidoreductase activity, acting on the CH-OH group of donors, NAD or NADP as acceptor |
| GO:0016614 | oxidoreductase activity, acting on CH-OH group of donors |
| GO:0004857 | enzyme inhibitor activity |
| GO:0016684 | oxidoreductase activity, acting on peroxide as acceptor |

Specific pathway information obtained from GO enrichment analysis of the transcriptome sequencing data.

**Supplementary Table5:**

**Abbreviations List**

| Abbreviations | Full Spelling |
| --- | --- |
| ECM | Extracellular Matrix |
| FBS | Fetal Bovine Serum |
| PEI | Polyethylenimine |
| SDS | Sodium Dodecyl Sulfate |
| BSA | Bovine Serum Albumin |

Information on abbreviations.
