# Supplementary material for: UHRF1-mediated HIF-1α stabilization promotes ovarian cancer through metabolic reprogramming and angiogenesis
Source: Cell Death Dis. 2025 Oct 24;16(1):751. doi: 10.1038/s41419-025-08033-w (PMC12552473; doi:10.1038/s41419-025-08033-w)
Supplement: Supplementary file 2 — Original western blots [file 41419_2025_8033_MOESM2_ESM.pdf]

F1-F

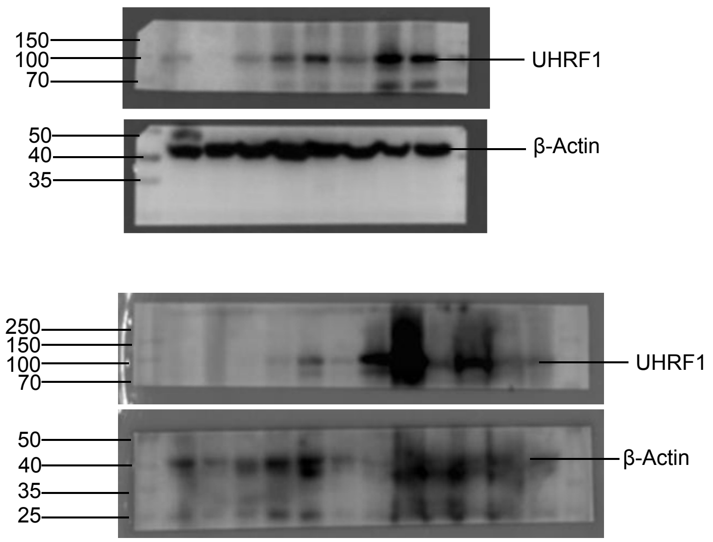

F1-G

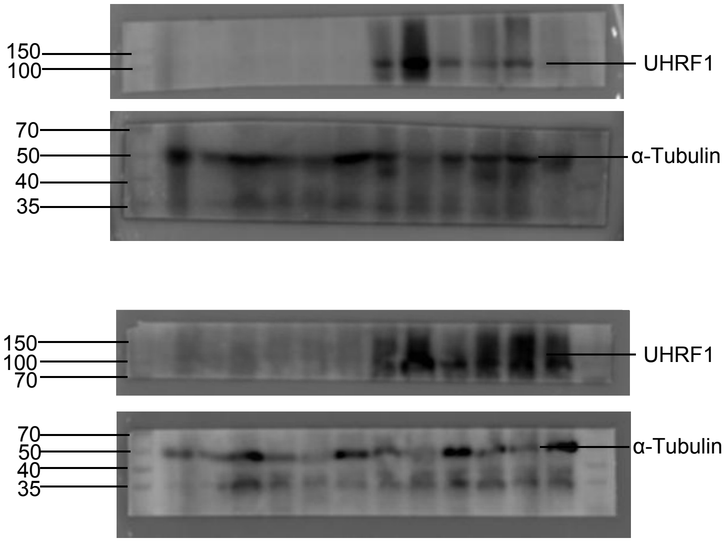

F4-C

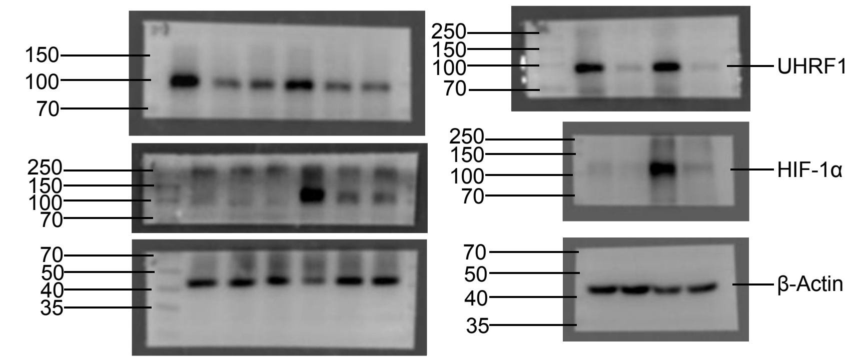

F4-D

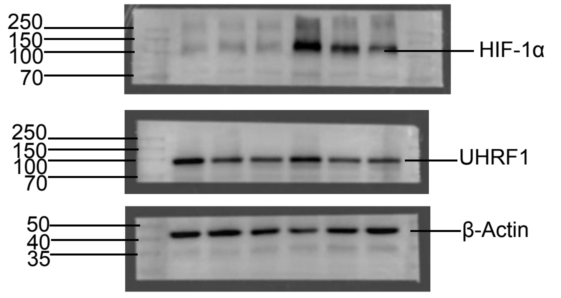

F4-E

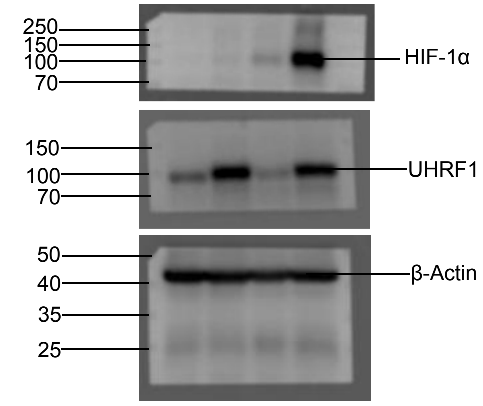

F4-G

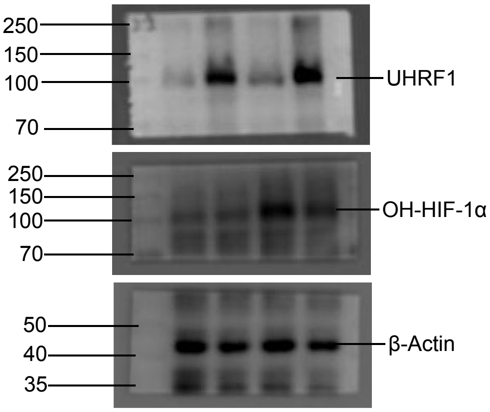

F4-H

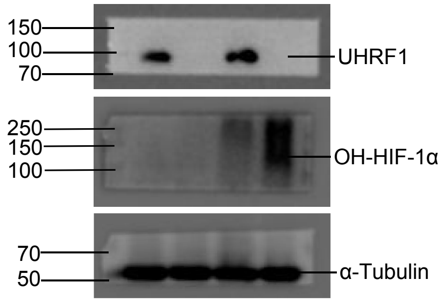

F4-I

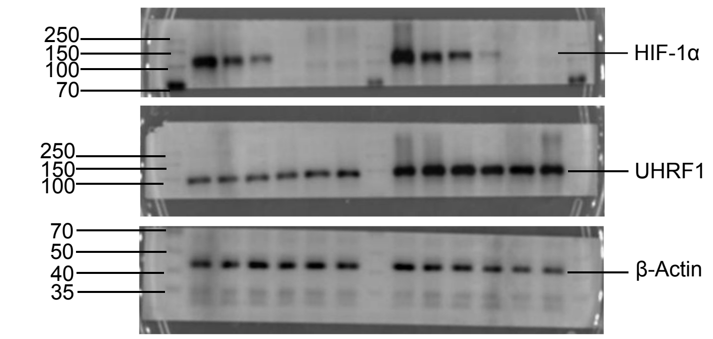

F4-J

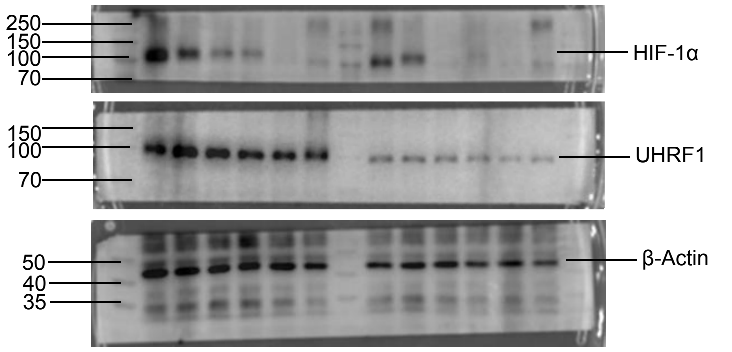

F4-M

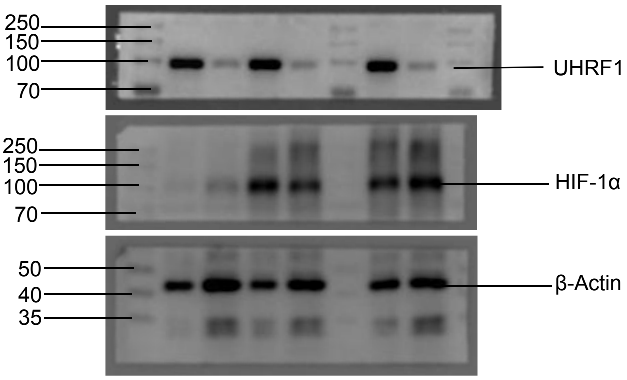

F4-N

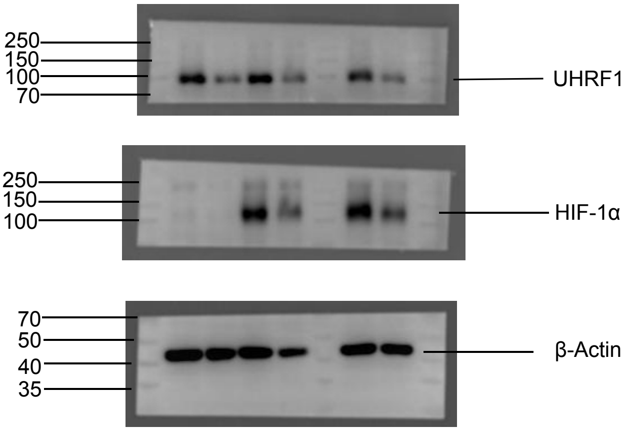

F4-P

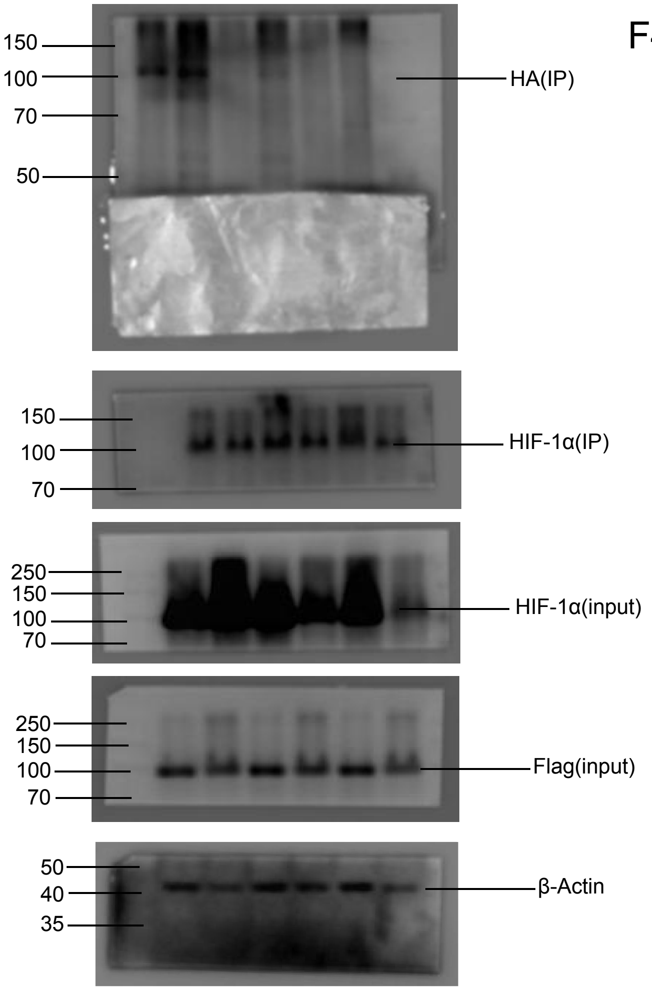

F4-O

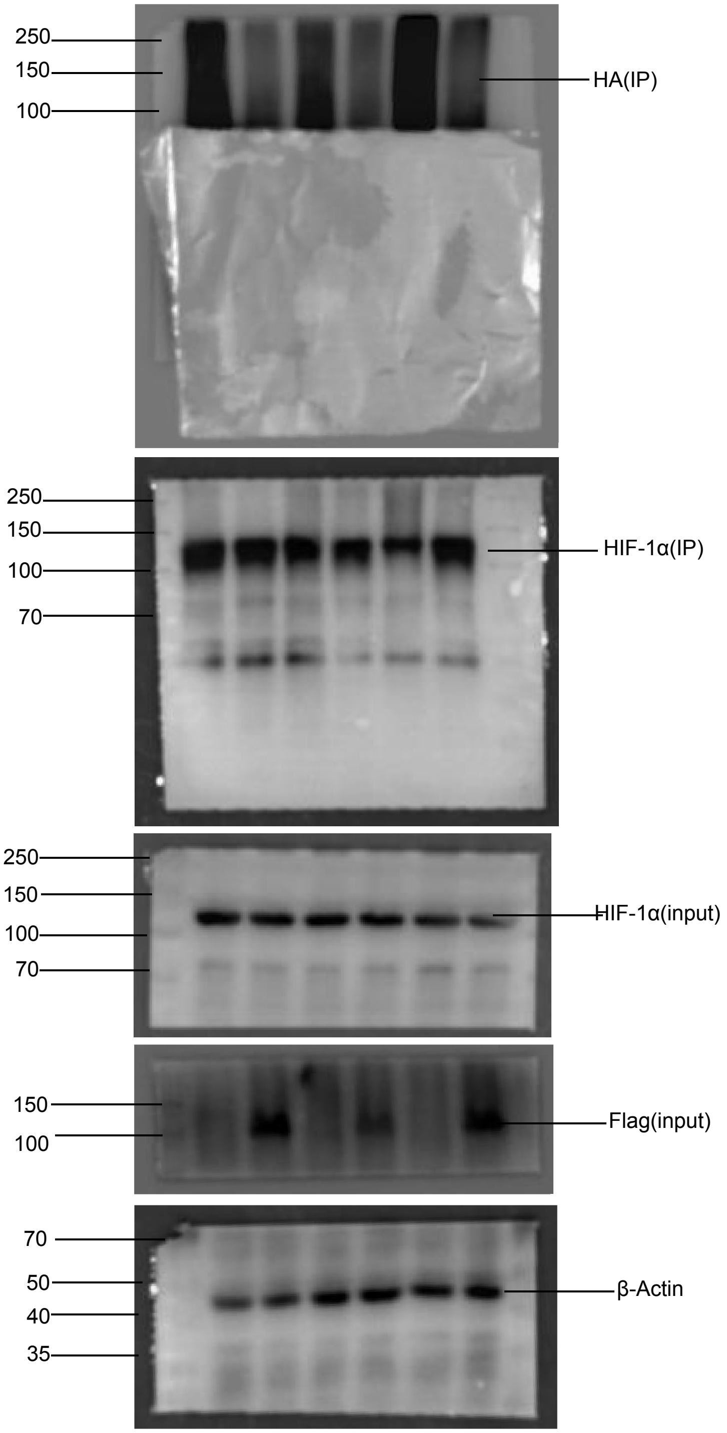

F5-C

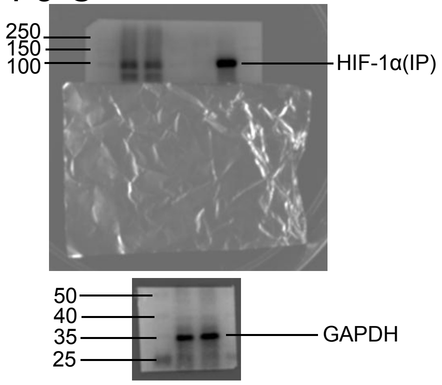

F5-D

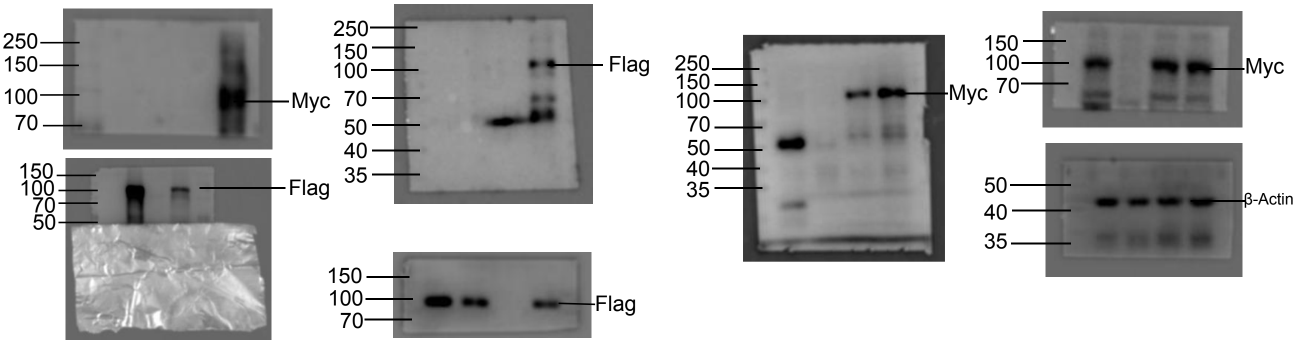

F5-E

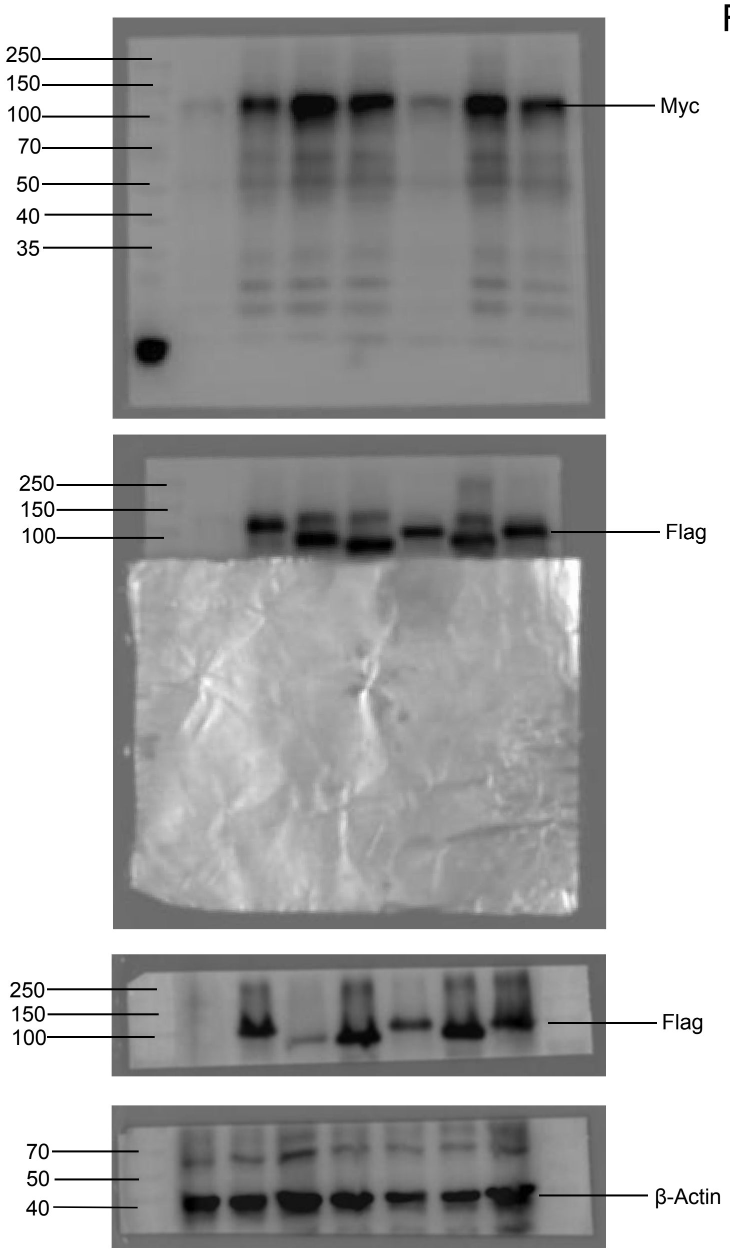

F5-H

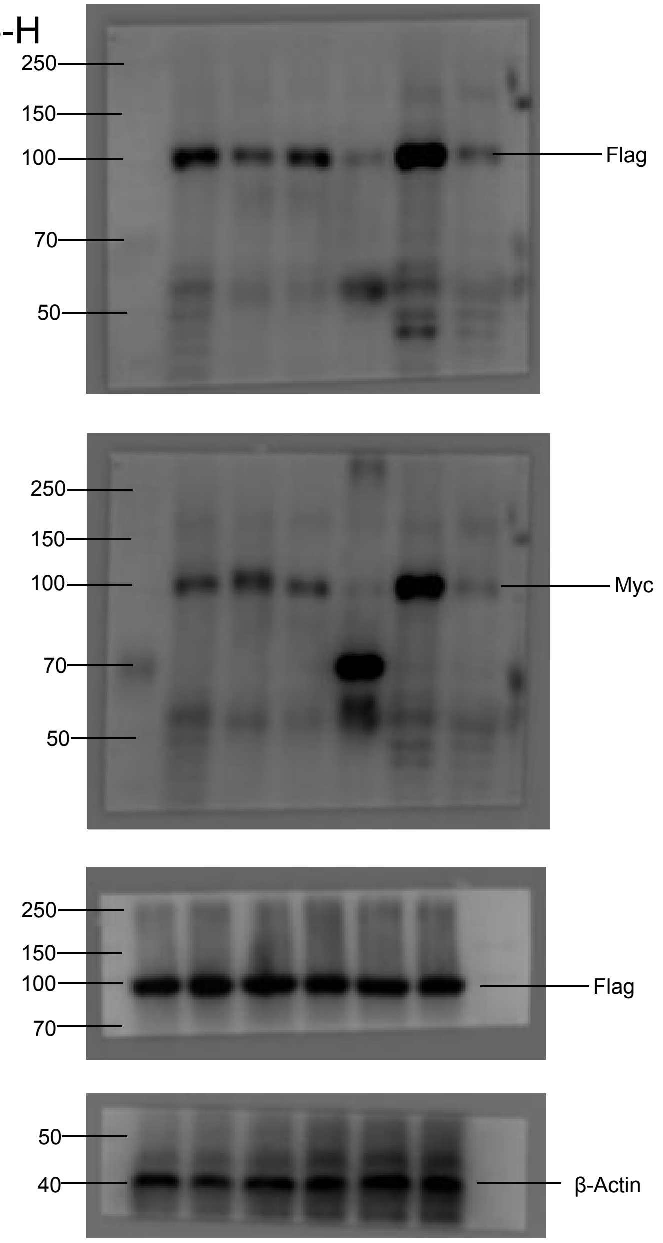

F5-I

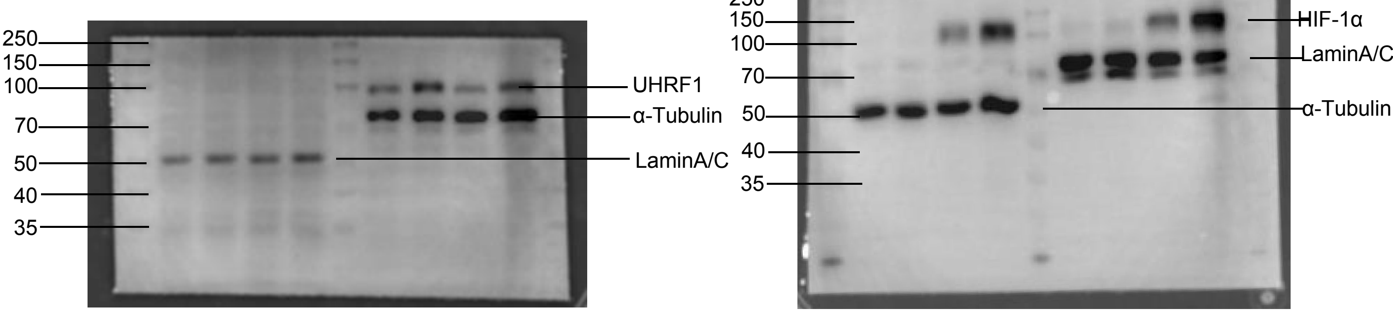

F6-H

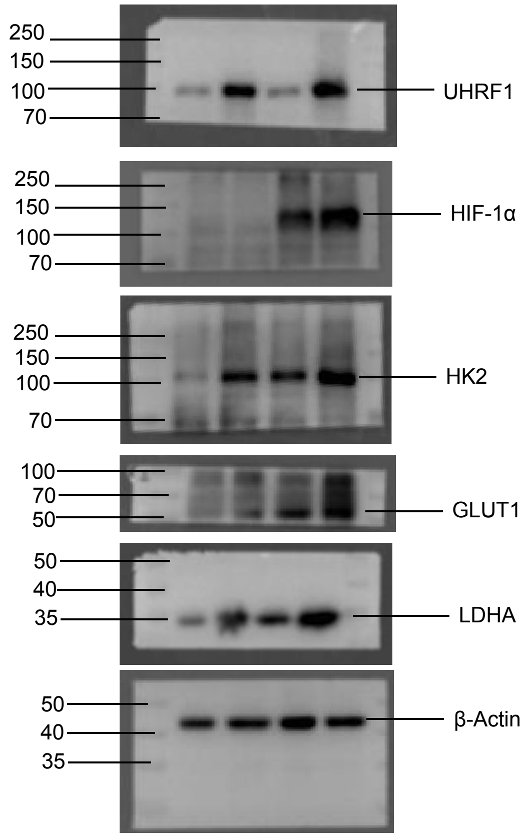

F6-I

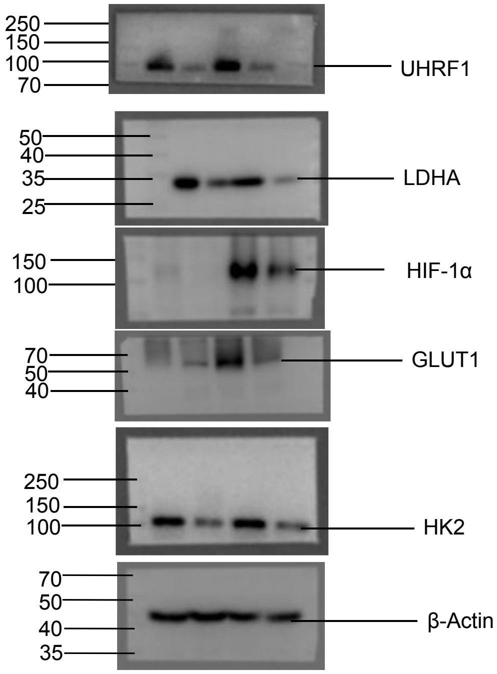

F7-C

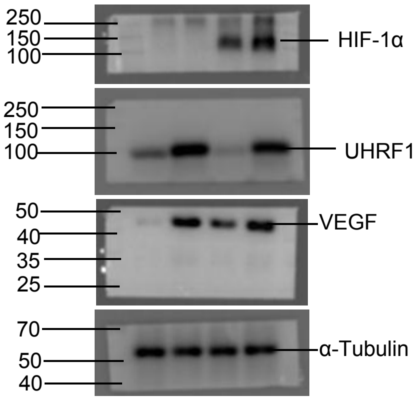

F7-D

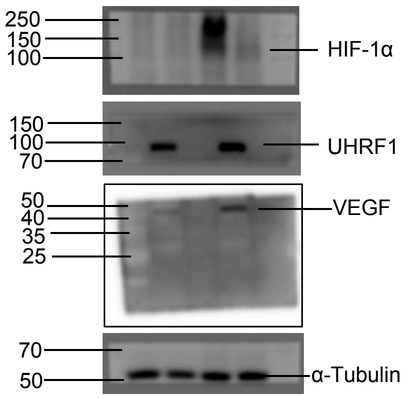

Sup-F2-A

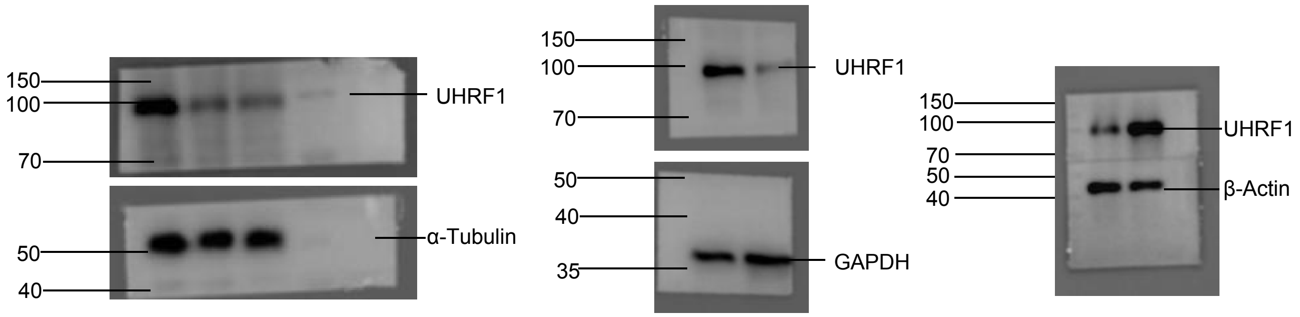

Sup-F2-B

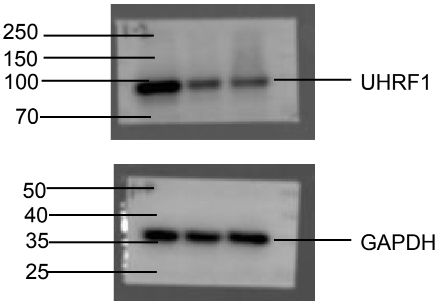

Sup-F2-H

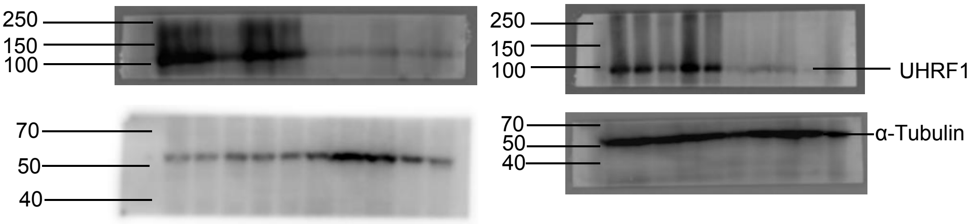

Sup-F3-A

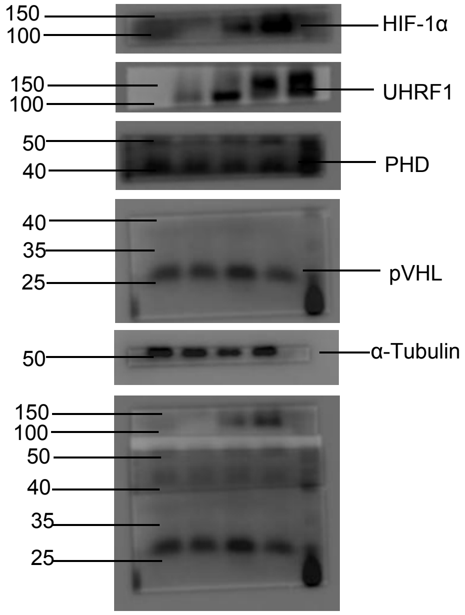

Sup-F3-B

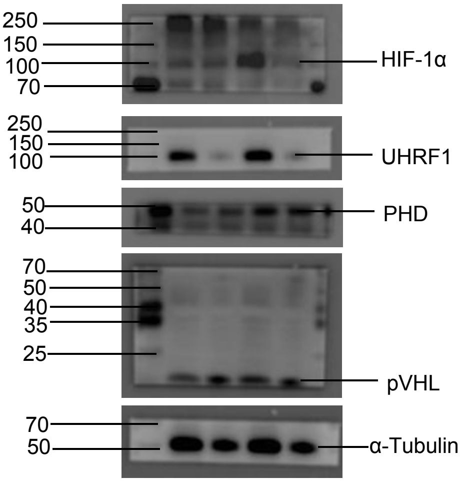

FS5-A

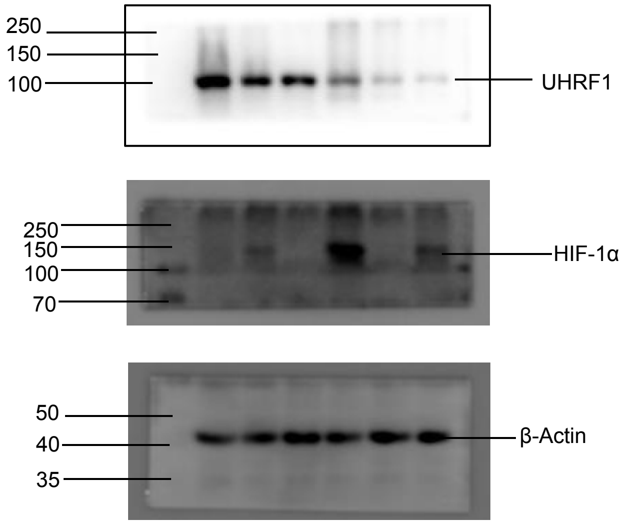

FS5-A

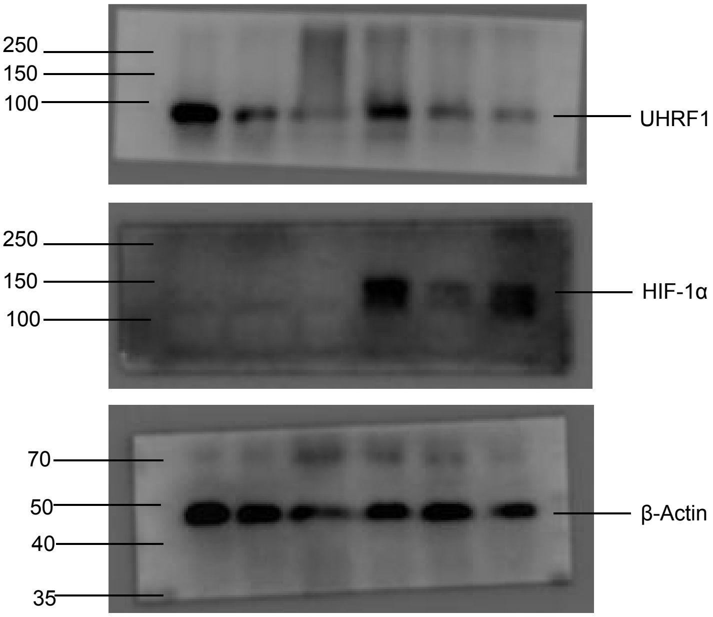

FS5-A

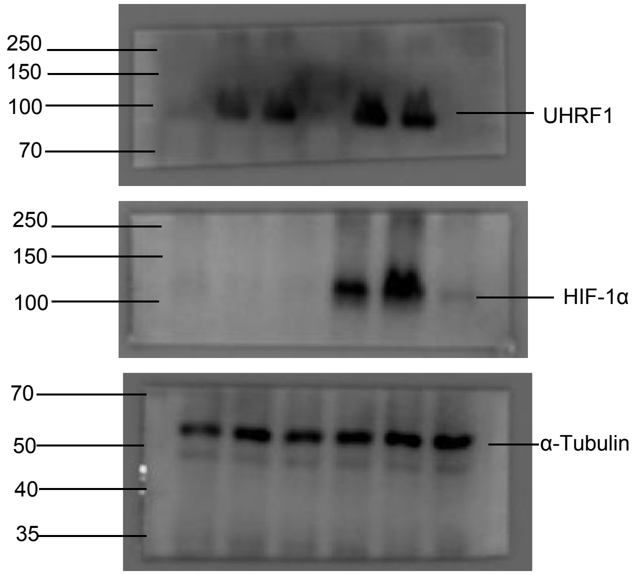
250
150
100
70
UHRF1
250
150
100
HIF-1α
70
50
40
35
α-Tubulin

FS5-B

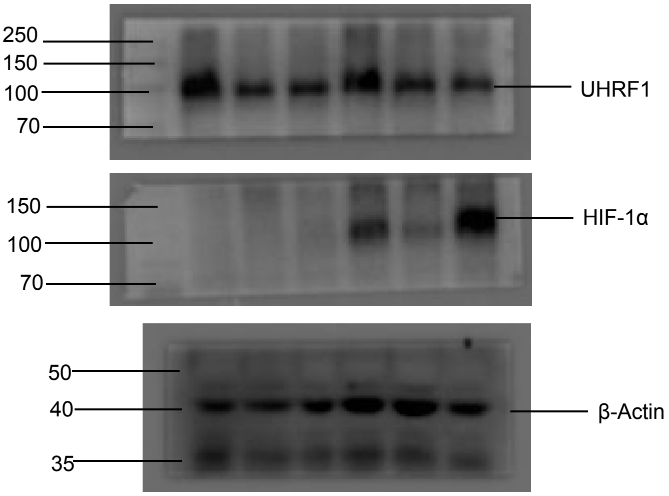
250
150
100
70
UHRF1
150
100
70
HIF-1α
50
40
35
β-Actin
